# Randomised trial of stable chest pain investigation: 3-year clinical and quality of life results from CE-MARC 2

**DOI:** 10.1136/openhrt-2022-002221

**Published:** 2023-05-02

**Authors:** Colin C. Everett, Colin Berry, Gerry P. McCann, Catherine Fernandez, Catherine Reynolds, Chiara Bucciarelli-Ducci, Erica Dall’Armellina, Abhiram Prasad, James R. Foley, Kenneth Mangion, Petra Bijsterveld, Julia Brown, Deborah Stocken, Simon Walker, Mark Sculpher, Sven Plein, John P. Greenwood

**Affiliations:** 1 School of Medicine, University of Leeds, Leeds, UK; 2 Institute of Cardiovascular and Medical Sciences, College of Medical, Veterinary and Life Sciences, University of Glasgow, Glasgow, UK; 3 Department of Cardiovascular Sciences, University of Leicester, Leicester, UK; 4 Department of Cardiology, Harefield Hospital, Harefield, UK; 5 Department of Cardiology, Leeds Teaching Hospitals NHS Trust, Leeds, UK; 6 Cardiovascular Diseases, Mayo Clinic Minnesota, Rochester, Minnesota, USA; 7 Centre for Health Economics, University of York, York, UK

**Keywords:** Magnetic Resonance Imaging, Tomography, Emission-Computed, Single-Photon, Angina Pectoris, Chest Pain, Coronary Artery Disease

## Abstract

**Aims:**

Guidelines for suspected cardiac chest pain have used historical risk stratification tools, advocating invasive coronary angiography (ICA) first-line in those at highest risk. We aimed to determine whether different strategies to manage suspected stable angina affected medium-term cardiovascular event rates and patient-reported quality of life (QoL) measures.

**Methods:**

CE-MARC 2, a three-arm parallel group trial, randomised patients with suspected stable cardiac chest pain and a Duke Clinical pretest likelihood of coronary artery disease between 10% and 90%. Patients were randomised to either first-line cardiovascular magnetic resonance (CMR), single-photon emission computed tomography (SPECT) or the UK National Institute for Health and Care Excellence (NICE) CG95 (2010) guidelines-directed care. For the three arms, 1-year and 3-year first major adverse cardiovascular event (MACE) rates and QoL assessed by the Seattle Angina Questionnaire, Short Form 12 (V.12) Questionnaire and EuroQol-5 Dimension Questionnaire were recorded.

**Results:**

1202 patients were randomised to CMR (n=481), SPECT (n=481) and NICE (n=240). Forty-two patients (18 CMR, 18 SPECT, 6 NICE) experienced one or more MACEs. The percentage rates (95% CIs) of MACE in the CMR, SPECT and NICE groups at 3 years were 3.7% (2.4%, 5.8%), 3.7% (2.4%, 5.8%) and 2.1% (0.9%, 4.8%), respectively. QoL scores did not significantly differ across domains.

**Conclusion:**

Despite a fourfold increase in referrals for ICA, the NICE CG95 (2010) guidelines risk-stratified care strategy did not significantly reduce 3-year MACE or improve QoL, as compared with functional imaging with CMR or SPECT.

**Trial registration number:**

ClinicalTrials.gov Registry (NCT01664858).

**WHAT IS ALREADY KNOWN ON THIS TOPIC**

- UK national guidelines on managing patients with stable chest pain (2010, CG95) recommended a risk-stratified management approach. However, if the risk model does not fit the local contemporary population, too many needless interventions (or too few necessary ones) may be produced, without any consequent downstream benefits in terms of quality of life or events avoided.

**WHAT THIS STUDY ADDS**

- Management by stress cardiovascular magnetic resonance (CMR) or single photon emission computed tomography (SPECT) as a first-line investigation resulted in fewer invasive angiograms within 12 months of randomisation than management following National Institute for Health and Care Excellence (NICE) guidelines CG95 (2010). Despite fewer angiograms, there was no significant difference in subsequent cardiovascular events at 3 years’ follow-up, and while some differences in quality of life domains were observed, the effects were small.

**HOW THIS STUDY MIGHT AFFECT RESEARCH, PRACTICE OR POLICY**

- Compared with NICE CG95 (2010), functional imaging first-line by CMR or SPECT resulted in significantly less-invasive procedures, but with no penalty in terms of 3-year major adverse cardiovascular event or quality of life outcome measures. The lack of difference in outcomes between CMR and SPECT suggests that a choice may be made between the two based on availability, cost, patient preference and shared decision-making.

## Background

Coronary artery disease (CAD) is a leading cause of death and disability worldwide. In secondary care, several non-invasive imaging tests are available to determine whether stable angina is due to obstructive CAD, which include CT coronary angiography (CTCA), myocardial perfusion scintigraphy by single-photon emission computed tomography (SPECT) and cardiovascular magnetic resonance (CMR).

In 2010, UK national guidelines on managing patients with stable chest pain recommended risk-stratified management.^1^ Using the Duke clinical risk score,^2^ participants with a pretest likelihood (PTL) of CAD of 10%–29% or 30%–60% were, respectively, recommended CTCA or functional imaging to decide the need for invasive coronary angiography (ICA). Those with PTL 61%–90% were recommended first-line ICA. This final aspect raised concerns, that already high rates of ICA would be increased further, since in contemporary practice, the Duke clinical risk score has been shown to overestimate CAD prevalence.^3^

The CE-MARC 2 trial^4^ showed that patient management by a uniform strategy of first-line CMR or SPECT resulted in fourfold fewer ICA procedures where no obstructive disease was evident, compared with the standard care strategy of National Institute for Health and Care Excellence (NICE) CG95 (2010)-based management, with no significant differences in major adverse cardiovascular event (MACE) rates after 1 year of follow-up.^5^ Prespecified secondary analyses of the trial were patient-reported quality of life (QoL) measures and medium-term cardiovascular outcomes at 3 years, which are reported here.

## Methods

### Trial design

CE-MARC 2 was a three-arm, parallel-group, multicentre, randomised, superiority trial of three distinct patient management strategies, the design of which has been previously published.^4 5^

### Patient and public involvement

Patient and public advisors were involved prior to the funding application in setting the trial research question, study design and outcome measures. They were also members of the trial management group and oversaw all aspects of trial delivery, and specifically reviewed all patient-facing trial documents.

### Participants

Patients were recruited from six UK secondary care rapid access chest pain clinics. After completing the baseline assessments, PTL of CAD based on the Duke clinical risk score was calculated to confirm eligibility, and to allow stratification.^3^ In brief, patients were eligible if they were aged ≥30 years, had stable suspected angina requiring further assessment, a defined Duke clinical PTL of CAD between 10% and 90%, were suitable for revascularisation if required and provided written informed consent. Exclusion criteria included non-anginal chest pain, a normal SPECT or CTCA result within the previous 2 years, being clinically unstable, previous myocardial infarction (MI), previous coronary revascularisation and contraindication to any study non-invasive imaging test.

### Interventions

Patients were randomised by minimisation (with age, sex, PTL category and recruiting site as balancing factors and a random element based on computer-generated random numbers) in a 2:2:1 ratio between CMR:SPECT:CG95 (2010)-directed care.^6^

- Patients randomised to CMR-guided care were scheduled for a CMR scan comprising left ventricle function, adenosine stress and rest perfusion imaging and late-gadolinium enhancement. Referral for ICA was indicated by an inconclusive or abnormal finding (two or more adjacent segments with 50% or more transmural extent of ischaemia, scar or ischaemia–scar combination) or where a normal finding was over-ruled by the treating clinician.^5^
- Patients randomised to SPECT-guided care were scheduled for SPECT imaging, comprising stress and rest studies carried out ideally within 5 days, using radioisotope traces 99mTc tetrofosmin/sestamibi, with stress imaging either by adenosine or using exercise. Referral for ICA was indicated by an inconclusive or abnormal finding (summed stress score ≥4), or where a normal finding was over-ruled by the treating clinician.^5^
- Standard care—following contemporary UK guidelines for chest pain of recent onset (CG95, 2010),^1^ participants were directed to one of three investigations, depending on the PTL of CAD calculated by site at baseline. Those with a calculated PTL of 61%–90% were directed to ICA. A PTL of 30%–60% led to a scheduled SPECT, in line with recommendations for functional cardiac imaging as a first-line test. A PTL of 10%–29% resulted in referral for CTCA, as per guidelines, where coronary artery calcium (CAC) scoring indicated one of either no further action (CAC score of 0), CTCA (CAC scores of 1–400) or referral for ICA (CAC scores over 400). Where CTCA was performed, a positive finding was any lesion of ≥50% in an epicardial coronary artery ≥2.5 mm in diameter. Referral for ICA was indicated by abnormal or inconclusive CTCA/SPECT findings, or normal findings over-ruled by the treating clinician.

### Outcome measures

The primary outcome measure was rates of ICA with no evidence of obstructive disease and has been published, along with the secondary outcome measure of rates of positive angiography and MACE within 12 months.^5^ MACE was defined as any cardiovascular death, non-fatal MI, unplanned revascularisation and hospitalisation for cardiovascular cause (acute coronary syndrome troponin negative, MI (types 1, 2, 4b), arrhythmia, stroke, heart failure; MI defined according to the third universal definition).^7^ An additional post-hoc clinical outcome measure of ‘hard event’ rate was defined as the time until first of cardiovascular death or MI.

Patient-reported QoL was measured using the Seattle Angina Questionnaire (SAQ) UK English, the Short Form 12 (V.12) Questionnaire (SF12v2), and the EuroQol 5-Dimension Questionnaire, 3 and 5 Levels (EQ-5D-3L and 5L), at randomisation, 6 months, 1 year, 2 years and 3 years; the validity and reliability of the 19-item SAQ, the SF12v2 and the EQ-5D have been previously demonstrated in cardiovascular studies (see online supplemental file 1 for details). Questionnaire scores were calculated according to scoring guidelines. For the SF12v2, we report the eight domain scales and two summary scores and the five domains of the SAQ.

10.1136/openhrt-2022-002221.supp1 Supplementary data

### Statistical methods

A sample size of 1200 patients provided at least 80% power for comparisons of the primary outcome measure.^4 5^ Allowing for 20% loss to follow-up, 1200 patients allowed us to estimate differences in 3-year MACE rates with the following precisions:

(1) CMR versus NICE would provide an estimate within ±3.9%–5.7%, assuming CMR to be 4% points greater than for NICE and 3-year NICE rates between 3% and 9%. (2) CMR versus SPECT would provide an estimate within ±3.3%–4.7%, assuming 4%-point difference and baseline 3-year rate of 3%–9%. Recruiting at least 50% of trial participants to the QoL substudy provides over 90% power (two-sided 5% significance level) to detect a clinically relevant difference of 10 points in the SAQ with an SD of 30. There were no formal interim analyses and no criteria for early trial termination.

Analysis was by intention-to-treat principles and comparisons of interest were NICE CG95 (2010) versus CMR and SPECT versus CMR. Time to first MACE was estimated by the Kaplan-Meier method, reporting proportion of patients with MACE at 1 and 3 years and univariate HR. The adjusted HR of risk of first MACE was estimated using Cox proportional hazard regression, adjusting for the four minimisation factors and also hypertension, smoking status and ethnicity.

The primary analysis of each QoL domain of the SAQ, SF12v2 and EQ-5D utilities was on a complete case basis including only questionnaires received and scored. Analyses were mixed-effects linear regression models of each scale score over time including the four minimisation factors, with fixed effects for baseline scale value, randomised arm, time and arm–time interaction, and random effects for patient and patient-time. A number of sensitivity analyses were included: (1) a fixed effect for baseline–time interaction, to allow for patients with higher scores at baseline having different trajectories to those with worse (lower) baseline scores; (2) ordinal proportional odds regression model to model the odds of having higher scores at follow-up in single-item scales; (3) a repeated measures covariance pattern model, replacing the assumption of linear changes over time, with that of a common unstructured correlation structure within each patient. Complete case analysis assumes the distribution of any missing data is the same as the observed data. Sensitivity analysis based on linear regression of imputed data was performed using multiple imputation by chained equations,^8^ 100 burn-in iterations with 60 fully imputed datasets (based on Fraction of Missing Information) created for each scale. Imputation was informed by minimisation factors and the following baseline variables: coronary artery bypass graft, percutaneous coronary intervention, angiogram, body mass index, vascular disease, cardiovascular disease, rheumatoid arthritis, beta-blocker use, statin, ACE inhibitor. No subgroup analyses were performed.

## Results

Between 23 November 2012 and 13 March 2015, 1202 participants were randomised to receive CMR-guided care (N=481), SPECT-guided care (N=481) or NICE CG95 (2010)-guided care (N=240). Over 97% of patients returned baseline QoL data. The flow of participants and their baseline clinical characteristics have been previously published.^5^ Table 1 presents a summary of demographic characteristics and baseline QoL data. There were no differences in baseline medication usage across the three trial arms (table 1). At 12 months, only statin therapy showed a statistically significant difference/change, with greater usage in the NICE arm compared with the CMR and SPECT arms (proportional net change +12.5%, +5.4%, +4.4%, respectively; Breslow-Day Χ^2^ 7.053, p=0.029).

**Table 1:** Patient characteristics and QoL scale scores at randomisation

	CMR-guided care (n=481)	SPECT-guided care (n=481)	NICE CG95 (2010) (n=240)	Total (n=1202)
Patient age (years), mean (SD)	56.5 (9.10)	55.9 (8.87)	56.5 (9.21)	56.3 (9.03)
Male	254 (52.8%)	256 (53.2%)	128 (53.3%)	638 (53.1%)
White	443 (92.1%)	443 (92.1%)	221 (92.1%)	1107 (92.1%)
Current smoker	123 (25.6%)	106 (22.0%)	65 (27.1%)	294 (24.5%)
Diabetic: type II	48 (10.0%)	64 (13.3%)	21 (8.8%)	133 (11.1%)
Hypertension	177 (36.8%)	182 (37.8%)	99 (41.3%)	458 (38.1%)
Family history of premature CHD	252 (52.4%)	259 (53.8%)	140 (58.3%)	651 (54.2%)
Body mass index, mean (SD)	29.2 (5.36)	29.1 (5.12)	29.0 (5.24)	29.1 (5.23)
Duke clinical PTL category (as randomised)				
10%–29%	128 (26.6%)	125 (26.0%)	61 (25.4%)	314 (26.1%)
30%–60%	179 (37.2%)	183 (38.0%)	88 (36.7%)	450 (37.4%)
61%–90%	174 (36.2%)	173 (36.0%)	91 (37.9%)	438 (36.4%)
Duke clinical PTL % (as analysed), mean (SD)	49.9 (24.25)	48.6 (23.57)	50.7 (23.28)	49.5 (23.78)
Baseline medication use				
Beta-blockers	150 (31.2%)	157 (32.6%)	74 (30.8%)	381 (31.7%)
Statins or other lipid-lowering medications	191 (39.7%)	201 (41.8%)	108 (45.0%)	500 (41.6%)
ACE inhibitor or angiotensin II receptor blocker	115 (23.9%)	122 (25.4%)	66 (27.5%)	303 (25.2%)
Antiplatelet agents	271 (56.3%)	268 (55.7%)	150 (62.5%)	689 (57.3%)
Other antianginal agents	283 (58.8%)	276 (57.4%)	142 (59.2%)	701 (58.3%)
SAQ-UK Angina Frequency score*	70.0 (50.0–80.0)	70.0 (60.0–80.0)	70.0 (50.0–80.0)	70.0 (50.0–80.0)
SAQ-UK Angina Stability score*	50.0 (25.0–50.0)	50.0 (25.0–50.0)	50.0 (25.0–50.0)	50.0 (25.0–50.0)
SAQ-UK Physical Limitation score*	77.8 (58.3–91.7)	75.0 (58.3–88.9)	77.8 (55.6–88.9)	77.8 (58.3–91.7)
SAQ-UK Quality of Life score*	50.0 (41.7–66.7)	50.0 (41.7–66.7)	50.0 (33.3–66.7)	50.0 (37.5–66.7)
SAQ-UK Treatment Satisfaction score*	100.0 (81.3–100.0)	100.0 (81.3–100.0)	93.8 (81.3–100.0)	100.0 (81.3–100.0)
SF12v2 Bodily Pain score†	75.0 (50.0–75.0)	75.0 (50.0–75.0)	75.0 (50.0–75.0)	75.0 (50.0–75.0)
SF12v2 General Health score†	60.0 (25.0–60.0)	60.0 (25.0–60.0)	60.0 (25.0–60.0)	60.0 (25.0–60.0)
SF12v2 Mental Health score†	62.5 (50.0–75.0)	62.5 (50.0–75.0)	62.5 (50.0–75.0)	62.5 (50.0–75.0)
SF12v2 Physical Function score†	50.0 (50.0–91.1)	50.0 (45.0–75.0)	50.0 (50.0–75.0)	50.0 (50.0–75.0)
SF12v2 Role Emotional score†	87.5 (62.5–100.0)	87.5 (50.0–100.0)	75.0 (50.0–100.0)	87.5 (62.5–100.0)
SF12v2 Role Performance score†	62.5 (50.0–87.5)	62.5 (50.0–75.0)	62.5 (50.0–87.5)	62.5 (50.0–78.9)
SF12v2 Social Functioning score†	75.0 (50.0–100.0)	75.0 (50.0–100.0)	75.0 (50.0–100.0)	75.0 (50.0–100.0)
SF12v2 Vitality score†	50.0 (25.0–75.0)	50.0 (25.0–75.0)	50.0 (25.0–75.0)	50.0 (25.0–75.0)
SF12v2 Physical Component score†	45.2 (37.4–51.7)	44.6 (37.8–50.9)	43.9 (38.5–51.4)	44.6 (37.8– 51.4)
SF12v2 Mental Component score†	50.7 (43.3–57.3)	50.5 (41.6–56.8)	49.6 (40.8–56.7)	50.5 (41.9–56.9)
EQ-5D-3L Utility‡	0.796 (0.691–0.883)	0.760 (0.689–0.848)	0.743 (0.656–0.883)	0.760 (0.689–0.850)
EQ-5D-5L Utility‡	0.879 (0.778–0.937)	0.859 (0.777–0.937)	0.859 (0.733–0.937)	0.861 (0.777–0.937)

Values are n (%), except where mean (SD) are stated and for the SAQ-UK, SF12v2 and EQ-5D values, for which median (lower quartile–upper quartile) are given. Further baseline characteristics are given in Greenwood *et al*.^5^

*Baseline SAQ reported by 1187 (99%) of 1202 patients.

†Baseline SF12 reported by 1192 (99%) of 1202 patients.

‡ED-5D baseline reported by 1168 (97%) of 1202 patients.

CHD, coronary heart disease; CMR, cardiovascular magnetic resonance; EQ-5D, EuroQol-5 Dimension Questionnaire; NICE, National Institute for Health and Care Excellence; PTL, pretest likelihood; QoL, quality of life; SAQ, Seattle Angina Questionnaire; SF12v2, Short Form 12 (V.12) Questionnaire; SPECT, single-photon emission computed tomography.

### Clinical events

**Figure 1:**
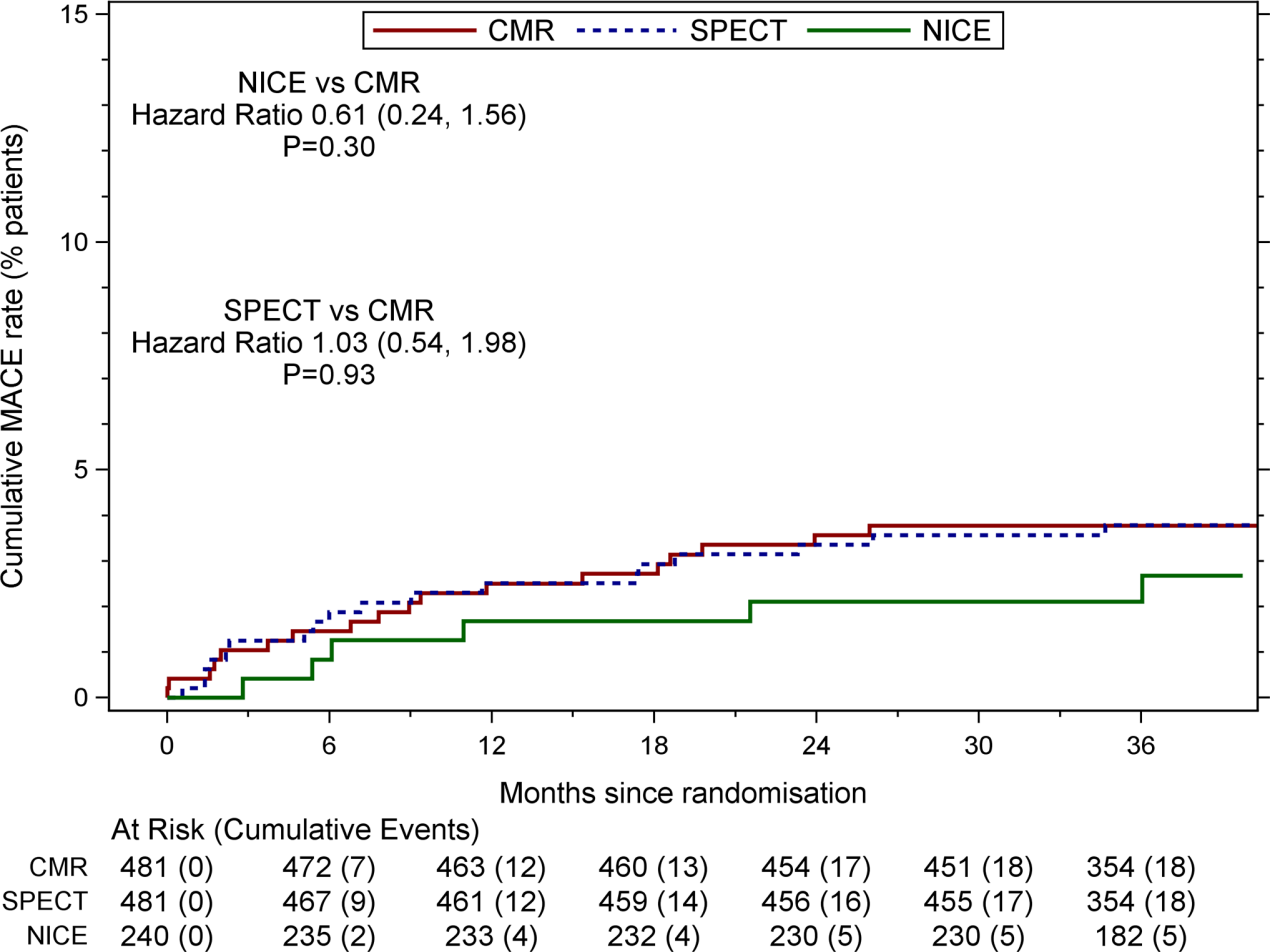
Kaplan-Meier estimates (with 95% CI) of time to first major adverse cardiovascular event (MACE) by arm. HRs are adjusted for randomising centre, sex, age category, pretest likelihood category, hypertension, smoking status and ethnicity (online supplemental appendix A). CMR, cardiovascular magnetic resonance; NICE, National Institute for Health and Care Excellence; SPECT, single-photon emission CT.

The annualised first MACE rate was low at 1.2% per year (table 2). Forty-two patients (18 CMR, 18 SPECT, 6 NICE) experienced 56 MACEs (26 CMR, 22 SPECT, 8 NICE). There was only a small absolute difference between the CMR and NICE arms in terms of 1-year and 3-year MACE, and no difference between the CMR and SPECT arms. The unadjusted HR of time to first MACE for NICE versus CMR was 0.66 (95% CI 0.26 to 1.67, p=0.38), and 1.00 (95% CI 0.52 to 1.92, p=1.00) for SPECT versus CMR. The adjusted HRs were similar to the unadjusted HRs (figure 1 and online supplemental appendix A). The 3-year ‘hard event’ rates were 1.5% (0.7% to 3.1%), 1.1% (0.4% to 2.5%) and 0.8% (0.2% to 3.3%) for CMR, SPECT and NICE, respectively.

**Table 2:** Summary of clinical outcomes

	CMR-guided care (n=481)	SPECT-guided care (n=481)	NICE CG95 (2010) (n=240)	Total (n=1202)
Number of events (number of patients)	26 (18)	22 (18)	8 (6)	56 (42)
Total follow-up (patient-years to first MACE or last contact)	1396.8	1397.6	704.3	3498.7
Annualised first MACE rate (%/year, 95% CI)	1.29 (0.78 to 1.98)	1.29 (0.78 to 1.98)	0.85 (0.34 to 1.73)	1.20 (0.87 to 1.60)
MACE within 1 year, % (95% CI)	2.5 (1.4 to 4.4)	2.5 (1.4 to 4.4)	1.7 (0.6 to 4.4)	
Absolute difference (comparator—CMR, 95% CI)	–	0.0 (−2.0 to 2.0)	−0.8 (−3.0 to 1.3)	
MACE within 3 years, % (95% CI)	3.8 (2.4 to 5.9)	3.8 (2.4 to 5.9)	2.1 (0.9 to 5.0)	
Absolute difference (comparator—CMR, 95% CI)	–	0.0 (−2.4 to 2.4)	−1.7 (−4.2 to 0.8)	
‘Hard event’ within 1 year, % (95% CI)	0.6 (0.2 to 1.9)	0.4 (0.1 to 1.7)	0.8 (0.2 to 3.3)	
Absolute difference (comparator—CMR, 95% CI)	–	−0.2 (−1.1 to 0.7)	0.2 (−1.1 to 1.6)	
‘Hard event’ within 3 years, % (95% CI)	1.5 (0.7 to 3.1)	1.1 (0.4 to 2.5)	0.8 (0.2 to 3.3)	
Absolute difference (comparator—CMR, 95% CI)	–	−0.4 (−1.8 to 1.0)	−0.6 (−2.2 to 1.0)	
Frequency of individual MACE events				
Type 3 MI and CV death: MI*	–	1	–	1
CV death: MI*	–	1	1	2
CV death: pulmonary embolism*	–	1	–	1
CV death: stroke*	1	–	–	1
CV death: unknown*	–	1	–	1
Unplanned PCI	7	5	2	14
Unplanned CABG	1	–	–	1
Type 1 MI*	7	1	2	10
Type 2 MI*	–	2	–	2
Arrhythmia	6	3	2	11
Stroke/TIA	4	3	–	7
Heart failure	–	4	1	5
Non-MACE event				
Non-CV deaths	7	1	2	10
Previously published findings^5^				
Primary outcome: unnecessary angiography within 12 months, n (%)	36 (7.5)	34 (7.1)	69 (28.8)	139 (11.6)
Secondary outcome: positive angiography within 12 months, n (%)	47 (9.8)	42 (8.7)	29 (12.1)	118 (9.8)

*Denotes an event count as part of the post-hoc ‘hard event’ outcome measure, comprising CV death and non-fatal MI (excluding periprocedural MI due to PCI or CABG).

CABG, coronary artery bypass graft; CMR, cardiovascular magnetic resonance; CV, cardiovascular; MACE, major adverse cardiovascular event; MI, myocardial infarction; NICE, National Institute for Health and Care Excellence; PCI, percutaneous coronary intervention; SPECT, single-photon emission CT; TIA, transient ischaemic attack.

### Quality of life

**Figure 2:**
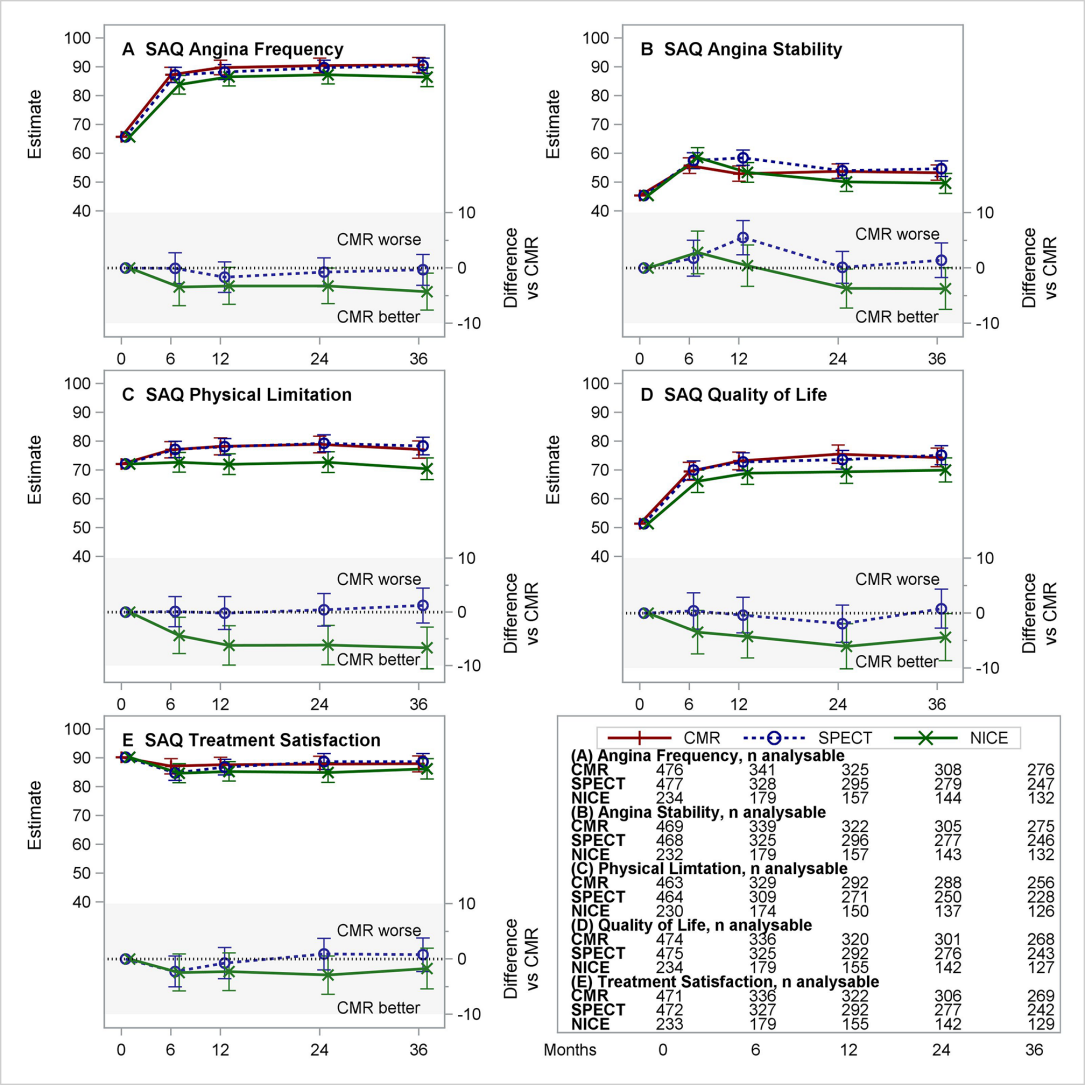
Estimated means for CMR, SPECT and NICE CG95 (2010) (and differences vs CMR) for the five domains of the SAQ. (A–E) Each panel presents the estimated least-squares means (with 95% CI) over time from repeated measures model for CMR, SPECT and NICE CG95 (2010)-based care (top section) and differences (with 95% CI) NICE–CMR and SPECT–CMR (lower section, shaded). Negative differences represent benefits for CMR versus comparator. Tables show the number of patients included from the complete case analysis. CMR, cardiovascular magnetic resonance; NICE, National Institute for Health and Care Excellence; SAQ, Seattle Angina Questionnaire; SPECT, single-photon emission CT.

**Figure 3:**
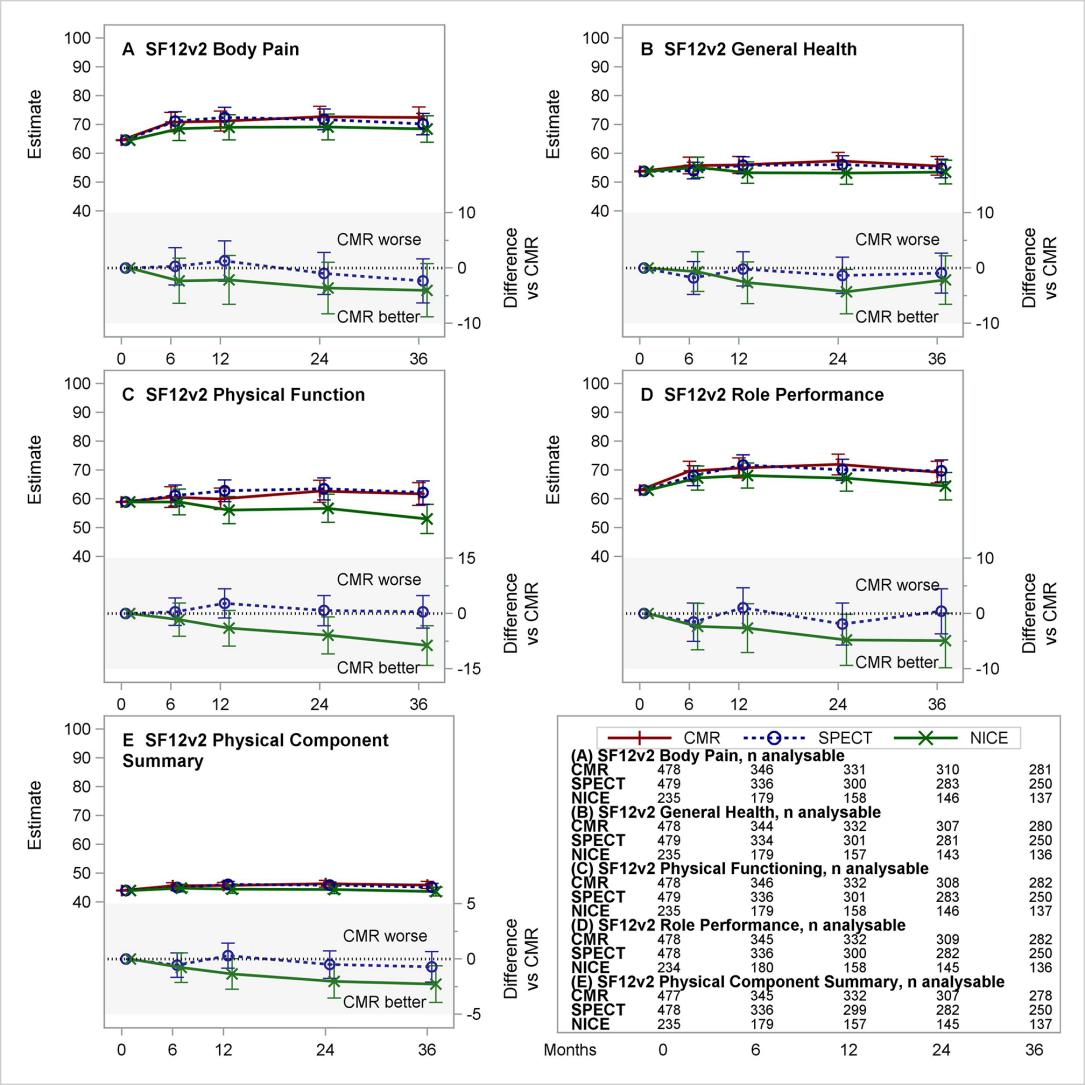
Estimated means for CMR, SPECT and NICE CG95 (2010) (and differences vs CMR) for the five physical domain and summary scores of the SF12v2. (A–E) Each panel presents the estimated least-squares means (with 95% CI) over time from repeated measures model for CMR, SPECT and NICE CG95 (2010)-based care (top section) and differences (with 95% CI) NICE–CMR and SPECT–CMR (lower section, shaded). Negative differences represent benefits for CMR versus comparator. Tables show the number of patients included from the complete case analysis. CMR, cardiovascular magnetic resonance; NICE, National Institute for Health and Care Excellence; SF12v2, Short Form 12 (V.2) Questionnaire; SPECT, single-photon emission CT.

**Figure 4:**
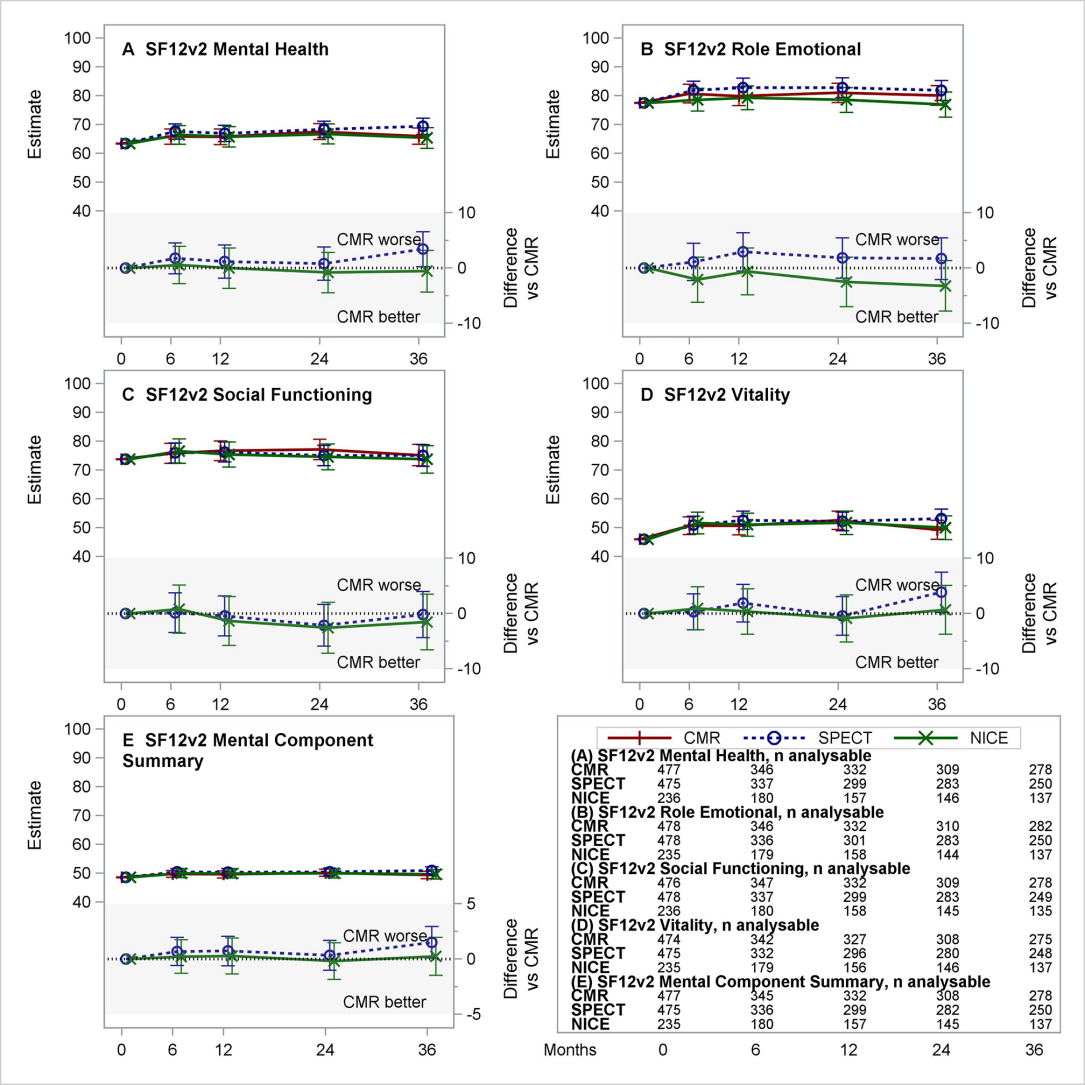
Estimated means for CMR, SPECT and NICE CG95 (2010) managed care (and differences vs CMR) for the five mental domain and summary scores of the SF12v2. (A–E) Each panel presents the estimated least-squares means (with 95% CI) over time from repeated measures model for CMR, SPECT and NICE CG95 (2010)-based care (top section) and differences (with 95% CI) NICE–CMR and SPECT–CMR (lower section, shaded). Negative differences represent benefits for CMR versus comparator. Table in lower right provides number of patients included from the complete case analysis. CMR, cardiovascular magnetic resonance; NICE, National Institute for Health and Care Excellence; SF12v2, Short Form 12 (V.2) Questionnaire; SPECT, single-photon emission CT.

**Figure 5:**
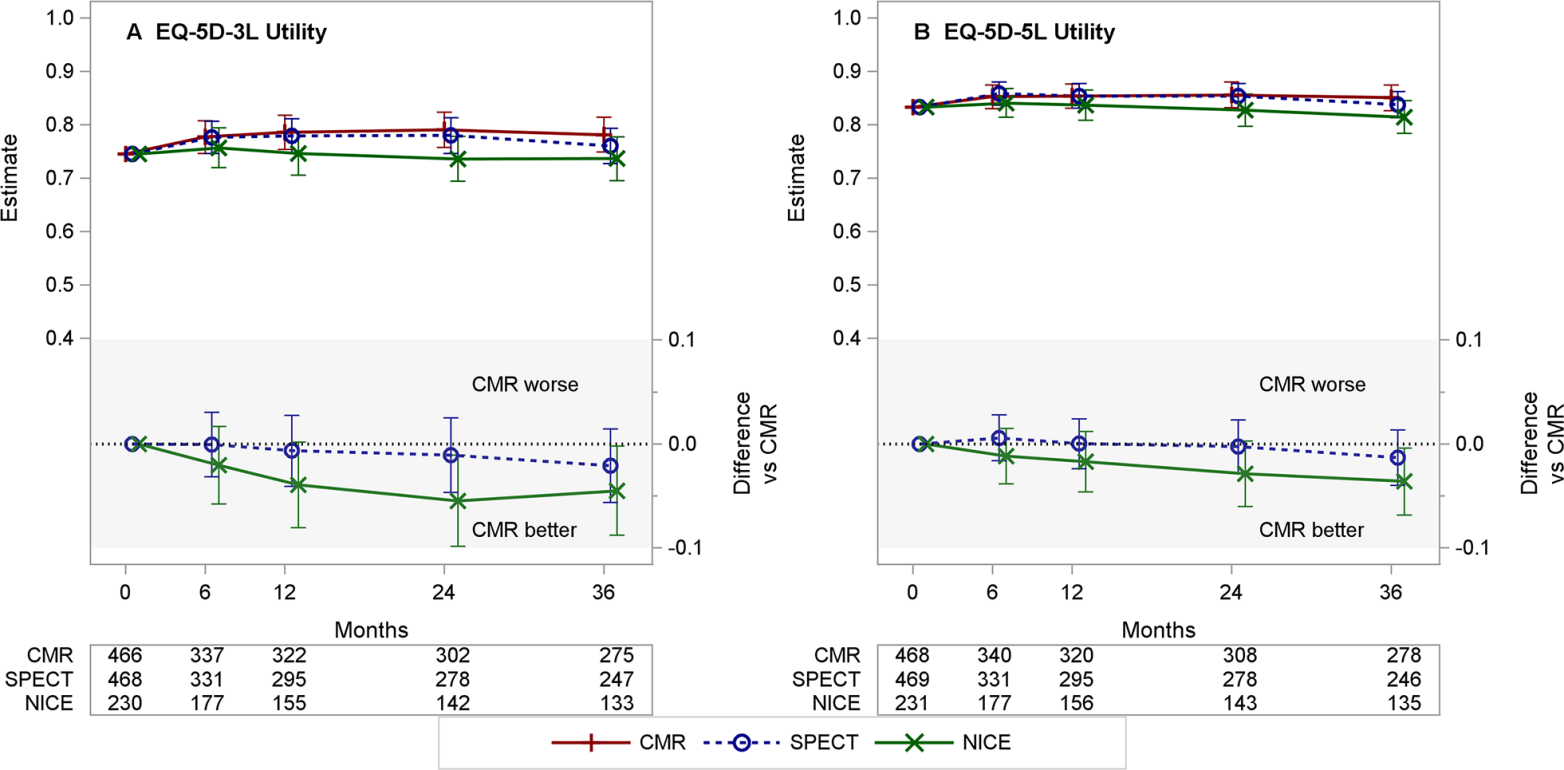
Estimated means for CMR, SPECT and NICE CG95 (2010) managed care (and differences vs CMR) for the EQ-5D-3L and EQ-5D-5L Utilities. (A, B) Each panel presents the estimated least-squares means (with 95% CI) over time from repeated measures model for CMR, SPECT and NICE CG95 (2010)-based care (top section) and differences (with 95% CI) NICE–CMR and SPECT–CMR (lower section, shaded). Negative differences represent benefits for CMR versus comparator. Table ishow the number of patients included from the complete case analysis. CMR, Cardiac Magnetic Resonance based care; EQ-5D-3L[−5 L], Euroqol 5-dimension questionnaire, 3 [5] levels; NICE, NICE CG95 (2010) based management; SPECT, Single Photon Emission CT based care.CMR, cardiovascular magnetic resonance; EQ-5D-3L/5L, EuroQol 5-Dimension Questionnaire, 3/5 Levels; NICE, National Institute for Health and Care Excellence; SPECT, single-photon emission CT.

Overall, 1168 (97%) of participants returned baseline questionnaire booklets at the point of randomisation. However, 670 (55%) of participants returned questionnaire booklets at the 3-year time point (annual returns listed for each questionnaire in figures 2–5), with 554 (46%) returning a complete set of questionnaire data at all five predefined time points (online supplemental appendix B). Online supplemental appendices report the frequency of floor and ceiling values and observed summary statistics for the SAQ (online supplemental appendix C), SF12v2 (online supplemental appendix D) and EQ-5D (online supplemental appendix E) by trial arm at each time point.

Table 3 summarises the intervention effect on the SAQ-UK scores over time based on complete case analysis. Figure 2 provides the estimated group means (and differences vs CMR) over time for the five domains of the SAQ-UK. Considering the multiplicity of comparisons, there was no apparent difference in scores over time between randomised treatment groups. Observed differences in the rates of change in QoL domains were small in relation to the range of the scale. The largest difference was estimated in the Angina Stability domain (estimate −0.224 points per month (95% CI −0.376 to −0.073)) and corresponds to a difference of 8 points (on a scale of 0–100) over a 3-year follow-up. The sensitivity analyses did not alter any of the findings or conclusions (online supplemental appendix C).

**Table 3:** Complete case analysis of QoL domains

Domain/analysis	CG95 (2010) vs CMR				SPECT vs CMR			
	Estimate	SE	95% CI		Estimate	SE	95% CI	
			Lower	Upper			Lower	Upper
Seattle Angina Questionnaire (UK version) (0=worst, 100=best)								
Angina Frequency	−0.023	0.056	−0.133	0.087	0.002	0.044	−0.084	0.088
Angina Stability*	−0.224	0.077	−0.376	−0.073	−0.080	0.064	−0.206	0.046
Physical Limitation	−0.072	0.055	−0.180	0.036	0.035	0.045	−0.054	0.124
Quality of Life	−0.053	0.068	−0.187	0.081	−0.004	0.054	−0.110	0.102
Treatment Satisfaction	0.010	0.060	−0.108	0.128	0.106	0.048	0.012	0.200
SF12v2 (0=worst, 100=best)								
Body Pain*	−0.078	0.070	−0.216	0.060	−0.106	0.059	−0.222	0.011
General Health*	−0.065	0.069	−0.200	0.071	0.005	0.056	−0.104	0.114
Physical Functioning	−0.224	0.083	−0.386	−0.062	−0.036	0.065	−0.164	0.093
Role Performance	−0.100	0.070	−0.238	0.039	0.019	0.059	−0.098	0.136
Physical Component Summary	−0.052	0.025	−0.101	−0.003	−0.016	0.021	−0.057	0.024
Mental Health	−0.043	0.063	−0.167	0.081	0.044	0.052	−0.058	0.146
Role Emotional	−0.061	0.071	−0.201	0.079	0.002	0.060	−0.116	0.121
Social Functioning*	−0.083	0.076	−0.232	0.067	−0.030	0.064	−0.156	0.097
Vitality*	−0.026	0.074	−0.171	0.120	0.074	0.060	−0.045	0.192
Mental Component Summary	−0.006	0.028	−0.061	0.050	0.020	0.023	−0.026	0.065
EQ-5D-3L (−0.594=worst, 1=perfect health)								
Utility^26^	−0.0009	0.0007	−0.0022	0.0004	−0.0007	0.0006	−0.0018	0.0004
EQ-5D-5L (−0.281=worst, 1=perfect health)								
Utility^27^	−0.0009	0.0005	−0.0019	0.0001	−0.0006	0.0004	−0.0014	0.0001

Estimates given are the arm–time interaction effects, estimating by how much the NICE CG95 and SPECT arm scores change per month, relative to those in the CMR arm. Negative values represent CMR getting better scores (vs comparator) over time, positive values represent CMR patients getting worse scores over time.

*This domain scale derives from a single question comprising five possible responses.

CMR, cardiovascular magnetic resonance; EQ-5D-3L/5L, EuroQol 5-Dimension Questionnaire, 3/5 Levels; NICE, National Institute for Health and Care Excellence; QoL, quality of life; SF12v2, Short Form 12 (V.2) Questionnaire; SPECT, single-photon emission CT.

The intervention effect on SF12v2 domain and summary scores over time, based on complete case analysis, are summarised in table 3. Figures 3 and 4 provide the estimated group means (and differences vs CMR) over time for the five SF12v2 Physical domains, the five SF12v2 Mental domains and summaries. Considering the multiplicity of comparisons, there were no apparent differences in scores over time between randomised treatment groups (table 3). Observed differences in the rates of change in QoL domains were small in relation to the range of the scale. The largest difference was estimated in the Physical Functioning domain (estimate −0.224 points per month (95% CI −0.386 to −0.062)) and corresponds to a difference of 8 points (on a scale of 0–100) over a 3-year follow-up. The sensitivity analyses did not alter any of the findings or conclusions (online supplemental appendix D).

Table 3 summarises the effect of intervention on the EQ-5D-3L and the EQ-5D-5L Utility scores, based on complete case analysis. Figure 5 provides the estimated group mean utility values (and differences vs CMR) over time. Although the estimated interaction effects for CMR versus NICE and for CMR versus SPECT both favoured the CMR arm, 95% CIs all enclosed the null value of zero indicating no significant difference. The sensitivity analyses did not alter any of the findings or conclusions (online supplemental appendix E).

Exploring patterns of missing data, the most consistent predictors of missing 36-month data were randomising centre, patient age, baseline scale values, current and prior use of beta-blockers, ACE inhibitors/angiotensin II receptor blockers and other antianginal medications; randomised allocation and PTL at randomisation were not.

## Discussion

The CE-MARC 2 trial compared three management strategies for secondary care patients with suspected stable angina. After the planned fixed 3 years’ follow-up of 1202 patients, there was no statistically significant difference in time to first MACE rates between the three arms of the trial. There were small numerical differences between trial arms, which differed in their pattern of clinical events, but which were too few to draw any inferences. In terms of ‘hard’ clinical events (death or MI), the rates were also comparable between the three trial arms; while again there were small numerical differences between trial arms, the study was not powered for this endpoint. Observed differences in QoL domains were small. MACE events and QoL were secondary outcome measures and these results supplement the main clinical trial findings,^5^ which showed a significantly higher rate of non-obstructive (‘unnecessary’) ICA findings for the NICE guidelines-based management strategy compared with the two functional imaging strategies, and only a small increase in positive detection of CAD.

We previously reported in high-risk patients with estimated PTL of 61%–90% of CAD, that the actual observed rate of disease was considerably lower than would be predicted, such that the odds of a non-obstructive ICA (or ‘unnecessary angiography’) for those randomised to NICE (CG95) guidelines-based management were 20 times greater than for those randomised to either CMR or SPECT-guided care.^5^ Since publication of NICE CG95 (2010), improved cardiovascular clinical prediction models have been proposed by the CAD Consortium and the PROMISE trial investigators.^3 9^ Both of these groups have developed models in much larger, more contemporary datasets. Despite this, implementation of these models into clinical practice without prior contemporary local^10^ recalibration for the population at risk may lead to the same outcome as in CE-MARC 2.

The near identical outcomes for CMR and SPECT patients were not expected. The similar specificities and superior sensitivities for CMR versus SPECT observed in the CE-MARC trial suggested we might see better disease detection in participants undergoing CMR, and so reduced MACE and better QoL.^11 12^ The numbers of patients undergoing ICA within 12 months in these two arms were similar and the numbers of patients with an ICA free from any obstructive disease were almost identical. Despite this, a greater proportion of SPECT patients had ICA due to a clinician referring for ICA despite a negative SPECT result, and a lower proportion of patients with a positive SPECT were subsequently referred for ICA, suggesting that overall clinician confidence in the SPECT result was lower than that for CMR.

Several randomised trials have evaluated non-invasive cardiac imaging for the management of patients with stable suspected cardiac chest pain, with predefined secondary endpoints of patient-reported QoL measures. These have predominantly involved CTCA versus standard care or versus functional testing (comprising a mixture of exercise ECG and functional imaging). The conclusions from the CE-MARC 2 QoL analysis are in line with other trials. The PROMISE trial randomised 10 003 patients to either functional testing or CTCA and found no difference in the composite outcome measure, EQ-5D-3L and SAQ after median 2.5 years’ follow-up.^13 14^ CRESCENT randomised 350 patients to CTCA or functional testing and found significant improvements in the SAQ Angina Frequency domain for CTCA versus functional testing at 12 months, but not in any other domain, or in EQ-5D or SF36.^15^ The follow-up CRESCENT-II trial of 268 patients found no differences in SAQ domain, EQ-5D or SF36 at 12 months.^16^ The SCOT-HEART trial of standard care (exercise stress ECG only and no additional testing) versus standard care+CTCA in 4146 patients reported, after median clinical follow-up of 4.8 years, an HR of 0.59 (95% CI 0.41 to 0.84) for the primary composite endpoint of CAD death or non-fatal MI in favour of standard care+CTCA.^17–19^ Despite a clinical strategy of coronary artery imaging for all patients, surprisingly, the CTCA strategy did not reduce the likelihood of undergoing ICA. Since CTCA involves ionising radiation, the strategy effectively doubles the number of tests involving ionising radiation in the referral population (most relevant in younger patients, especially females). The patient population for SCOT-HEART appears to have similar baseline SAQ scores and clinical characteristics to CE-MARC 2. However, in SCOT-HEART, CTCA was associated with less improvements in physical limitation, angina frequency and QoL at 6 months compared with standard care alone,^18^ though these absolute differences were small (<5-point difference) and no adjustment for multiple comparisons was performed. The reasons for this finding are complex, but likely include the possibility that an increase in the diagnosis of mild and moderate non-obstructive CAD detected by CTCA labels the patient with a life-long medical condition, creating anxiety and stress. Furthermore, in SCOT-HEART, medication for symptoms was discontinued in patients with no obstructive CAD, and this may have led to a deterioration in symptoms and QoL in patients with microvascular angina and/or vasospastic angina. This is one reason that the term ‘unnecessary angiography’ is no longer favoured, as the cardiology community embrace the more inclusive description of ischaemia with non-obstructive coronary arteries.

In terms of other CMR trials, the recently published MAGNET trial randomised 200 patients to either first-line ICA or first-line CMR, finding no significant difference at 3 years in the composite outcome of cardiac death and MI, despite a large reduction in revascularisation among those undergoing CMR.^20^ At 3-year follow-up, no between-arm differences were observed in any SAQ domain, though the CMR-guided group were reported to have higher domain scores at 1 year. Finally, MR-INFORM randomised 918 patients to a revascularisation strategy guided either by CMR or by ICA±fractional flow reserve measurement. Randomisation to CMR-guided care resulted in a lower rate of ICA, without an increase in the 1-year rate of composite cardiovascular outcome measure of all-cause death, non-fatal MI or revascularisation.^21^ Although EQ-5D was collected as part of this trial, no QoL data have been published yet.

### Limitations

The CE-MARC 2 trial population was predominantly white northern European, potentially limiting generalisability to other populations.^5^ Guidelines-based management relied on the Duke clinical risk score,^22^ a validated score used in the American Heart Association/American College of Cardiology guidelines^23^ at the time of trial recruitment, but which has since been reported to overestimate the PTL of CAD in contemporary trial populations.^3^ While the NICE CG95 guidelines were updated in 2016 to recommend CTCA as the initial investigation for all patients with de-novo atypical or typical angina, the European Society of Cardiology 2019 guidelines for the diagnosis and management of chronic coronary syndromes^24^ still recommend a range of initial investigations dependent on estimated PTL. This can include anatomical or functional imaging tests for patients at lower to intermediate risk, but also direct to ICA for those with high PTL, much like the original NICE CG95 (2010) guidelines evaluated in this trial.

While the observed annualised event rate of 1.2% per year was lower than anticipated, it was in line with published stable CAD trials such as SCOT-HEART,^17^ and provided an important safety outcome measure for the trial. Although the rate of MACE was lower for NICE CG95 (2010) versus CMR patients, the low event rate meant that even a large reduction in risk (adjusted HR of 0.66) did not conclude superiority for NICE versus CMR. Despite this limitation, CE-MARC 2 is a high-quality dataset which contributes importantly to the clinical evidence base and future meta-analysis.^25^

The validity of our QoL analyses relies on two key assumptions. The first relates to the unobserved data due to non-response. In CE-MARC 2, 55% of patients had analysable 3-year follow-up scale values (46% returned all five questionnaire booklets) and provided a powered complete case analysis. The primary complete case analysis included all observed data in a mixed-effects model under the assumption that data were missing at random. This model included the effects of the randomising site, baseline scale value and patient age, which were found to be consistently predictive of missing data, suggesting that the assumption was reasonable. A planned sensitivity analysis based on multiply imputed datasets^8^ produced similar results, and did not alter our conclusions.

The second assumption was that the sample size was sufficiently large that the distribution of the sample means would be normally distributed. Due to the lower-than-expected patient risk profile, observed scale values suffered from ceiling effects. At a number of time points, some domain scores had distributions comprising 25%–50% ceiling values. The distribution of the QoL domain scale scores in CE-MARC 2 raises questions as to the utility of these scales in this population. Comparable trials reported similarly skewed distributions in their QoL outcomes.^14 18^ Additionally, a large validation study of the original SAQ indicated pronounced ceiling effects in stable CAD for four of the five SAQ domain scores. Questionnaires may need to be refined to be more sensitive to change in this patient group.

## Conclusion

Despite a fourfold increase in referrals for ICA, the NICE CG95 (2010) guidelines risk-stratified care strategy did not reduce 3-year MACE or improve QoL, as compared with functional imaging with CMR or SPECT.

## Data Availability

Data are available upon reasonable request.
