# Supplementary material for: Randomised trial of stable chest pain investigation: 3-year clinical and quality of life results from CE-MARC 2
Source: Open Heart. 2023 May 2;10(1):e002221. doi: 10.1136/openhrt-2022-002221 (PMC10163591; doi:10.1136/openhrt-2022-002221)
Supplement: Supplementary data [file openhrt-2022-002221supp001.pdf]

Supplementary Appendix

Randomised trial of stable chest pain investigation (CE-MARC 2): 3-year clinical and quality of life results

Content

Questionnaire choice, validation and reliability

Supplemental Appendix A: Questionnaire completion.

Supplemental Appendix B: Supplemental results for Seattle Angina Questionnaire (UK) analyses.

Supplemental Appendix C: Supplemental results for SF12v2 analyses.

Supplemental Appendix D: Supplemental results for EuroQol EQ-5D analyses.

Supplemental Appendix E: Unadjusted and Adjusted Hazard Ratios, with full regression model results

Questionnaire choice, validation and reliability

Patient-reported QoL was measured using the Seattle Angina Questionnaire (SAQ) UK English,(1) the SF12v2 (2) and the Euroqol EQ-5D-3L (3) and -5L (4) at randomisation, 6 months, 1 year, 2 years and 3 years. The validity and reliability of the 19-item SAQ (1,5), the SF12 (6) and the EQ-5D (7,8) has been previously demonstrated in cardiovascular studies

References:

1. Spertus JA, Winder JA, Dewhurst TA et al. Development and evaluation of the Seattle Angina questionnaire: A new functional status measure for coronary artery disease. Journal of the American College of Cardiology 1995;25:333-341.

2. Ware JE, Kosinski M, Keller SD. A 12-Item Short-Form Health Survey: Construction of Scales and Preliminary Tests of Reliability and Validity. Medical Care 1996;34:220-233.

3. The EuroQol Health Policy Group. EuroQol-a new facility for the measurement of health-related quality of life. 1990;16:199-208.

4. Herdman M, Gudex C, Lloyd A et al. Development and preliminary testing of the new five-level version of EQ-5D (EQ-5D-5L). 2011;20:1727-1736.

5. Patel KK, Arnold SV, Chan PS et al. Validation of the Seattle angina questionnaire in women with ischemic heart disease. American Heart Journal 2018;201:117-123.

6. Failde I, Medina P, Ramirez C, Arana R. Construct and criterion validity of the SF-12 health questionnaire in patients with acute myocardial infarction and unstable angina. Journal of Evaluation in Clinical Practice 2010;16:569-573.

7. Wu J, Han YR, Zhao FL, Zhou J, Chen ZJ, Sun H. Validation and comparison of EuroQoL-5 dimension (EQ-5D) and Short Form-6 dimension (SF-6D) among stable angina patients. Health and Quality of Life Outcomes 2014;12.

8. Golicki D, Niewada M, Buczek J et al. Validity of EQ-5D-5L in stroke. Quality of Life Research 2015;24:845-850.

Supplemental Appendix A:

Tables provide the unadjusted and adjusted hazard ratios for time to first Major Adverse Cardiovascular Event (MACE) for the NICE vs CMR comparison, and for the SPECT vs CMR comparison. For the multivariable models, the full model fits with adjusted hazard ratios are given, except where estimation is not feasible due to small numbers. Categorical prognostic factors with 3 or more levels do not include P-Values for each level, rather the overall effect.

In all tables, a hazard ratio greater than 1 indicates increased risk of having a MACE (or experiencing MACE sooner) compared to the reference category level.

Table A.1: Unadjusted univariable hazard ratios for MACE for covariates in all 1,202 participants

Effect	Hazard Ratio (95% CI)	P-Value
Randomising Centre	Likelihood Ratio Chi-Sq: X^2 (5 df) = 1.612	0.900
Sex (MALE vs Female)	0.90 (0.49 to 1.64)	0.721
Age Group (65Y OR OLDER vs Under 65)	1.48 (0.75 to 2.95)	0.278
Pre-Test Likelihood category (Overall Effect)	Likelihood Ratio Chi-Sq: X^2 (4 df) = 6.427	0.169
Hypertension (YES vs No)	2.21 (1.20 to 4.07)	0.011
Smoking Status (Overall effect)	Likelihood Ratio Chi-Sq: X^2 (2 df) = 4.745	0.093
Ethnicity (NON-WHITE vs White)	1.25 (0.45 to 3.50)	0.682
Type II Diabetes (YES vs No+T1)	1.64 (0.73 to 3.70)	0.257
Body Mass Index (per kg/m2)	1.01 (0.96 to 1.07)	0.682

Note: all covariates except Type II diabetes and Body Mass Index were pre-specified as being included in the analysis Type II diabetes and Body Mass Index are post hoc analyses presented in response to manuscript review and are not included in subsequent tables. Note the adjustment for PTL category includes adjustment for the effect of diabetes on outcome.

Table A.2: Univariable and Full Model Fit results for comparison of NICE CG95 (2010) vs CMR (NICE and CMR participants only)

Effect	Hazard Ratio (95% CI)	P-Value
**Univariable: Unadjusted**		
NICE CG95 (2010) vs 3T-CMR	0.66 (0.26, 1.67)	P=0.38
**Multivariable: Adjusted**		
NICE CG95 (2010) vs 3T-CMR	0.61 (0.24, 1.56)	P = 0.303
Randomising Centre (Overall effect)	Likelihood Ratio Chi-Sq: X^2 (5df) = 6.93	P = 0.226
Sex: MALE vs Female	0.51 (0.20, 1.28)	P = 0.151
Age Group: 65Y OR OLDER vs Under 65	1.38 (0.47, 4.09)	P = 0.557
Pre-test Likelihood category (Overall effect)	Likelihood Ratio Chi-Sq: X^2 (4df) = 6.25	P = 0.181
PTL: 0-9% vs 10-29%	-	
PTL: 30-60% vs 10-29%	4.32 (0.92, 20.30)	
PTL: 61-90% vs 10-29%	4.01 (0.77, 20.94)	
PTL: 91-100% vs 10-29%	-	
Hypertension: YES vs No	2.38 (1.00, 5.68)	P = 0.051
Smoking Status (Overall effect)	Likelihood Ratio Chi-Sq: X^2 (2df) = 4.19	P = 0.123
Smoking status: EX vs Never	1.48 (0.50, 4.35)	
Smoking status: CURRENT vs Never	2.95 (1.01, 8.58)	
Ethnicity: NON-WHITE vs White	2.49 (0.60, 10.27)	P = 0.208

Table A.3: Univariable and Full Model Fit results for comparison of CMR vs SPECT (CMR and SPECT participants only)

Effect	Hazard Ratio (95% CI)	P-Value
**Univariable: Unadjusted**		
SPECT vs 3T-CMR	1.00 (0.52, 1.92)	P=1.00
**Multivariable: Unadjusted**		
3T-CMR vs SPECT	1.03 (0.54, 1.98)	P = 0.928
Randomising Centre (Overall effect)	Likelihood Ratio Chi-Sq: X^2 (5df) = 0.47	P = 0.993
Sex: MALE vs Female	0.80 (0.36, 1.78)	P = 0.593
Age Group: 65Y OR OLDER vs Under 65	1.32 (0.54, 3.22)	P = 0.547
Pre-test Likelihood category (Overall effect)	Likelihood Ratio Chi-Sq: X^2 (4df) = 3.38	P = 0.496
PTL: 0-9% vs 10-29%	-	
PTL: 30-60% vs 10-29%	1.24 (0.44, 3.55)	
PTL: 61-90% vs 10-29%	2.32 (0.79, 6.80)	
PTL: 91-100% vs 10-29%	1.43 (0.16, 13.11)	
Hypertension: YES vs No	2.20 (1.11, 4.38)	P = 0.025
Smoking Status (Overall effect)	Likelihood Ratio Chi-Sq: X^2 (2df) = 4.95	P = 0.084
Smoking status: EX vs Never	1.98 (0.87, 4.53)	
Smoking status: CURRENT vs Never	2.62 (1.04, 6.59)	
Ethnicity: NON-WHITE vs White	1.69 (0.55, 5.15)	P = 0.356

Appendix B: Completion of questionnaires

Table B.1 Returned questionnaire booklets

	3T CMR-Guided Care (n=481)	SPECT-Guided Care (n=481)	NICE CG95 (2010) (n=240)	Total (n=1202)
**Baseline**				
Not completed	3 (0.6%)	2 (0.4%)	4 (1.7%)	9 (0.7%)
In clinic	472 (98.1%)	474 (98.5%)	233 (97.1%)	1179 (98.1%)
Home (Postal)	2 (0.4%)	4 (0.8%)	2 (0.8%)	8 (0.7%)
Home (Telephone)	4 (0.8%)	1 (0.2%)	1 (0.4%)	6 (0.5%)
**6 months**				
Not completed	133 (27.7%)	144 (29.9%)	60 (25.0%)	337 (28.0%)
In clinic	1 (0.2%)	2 (0.4%)	-	3 (0.2%)
Home (Postal)	343 (71.3%)	326 (67.8%)	177 (73.8%)	846 (70.4%)
Home (Telephone)	4 (0.8%)	9 (1.9%)	3 (1.3%)	16 (1.3%)
**12 months**				
Not completed	149 (31.0%)	180 (37.4%)	82 (34.2%)	411 (34.2%)
In clinic	1 (0.2%)	1 (0.2%)	-	2 (0.2%)
Home (Postal)	323 (67.2%)	292 (60.7%)	155 (64.6%)	770 (64.1%)
Home (Telephone)	8 (1.7%)	8 (1.7%)	3 (1.3%)	19 (1.6%)
**24 months**				
Not completed	171 (35.6%)	197 (41.0%)	94 (39.2%)	462 (38.4%)
In clinic	2 (0.4%)	1 (0.2%)	-	3 (0.2%)
Home (Postal)	305 (63.4%)	279 (58.0%)	144 (60.0%)	728 (60.6%)
Home (Telephone)	3 (0.6%)	4 (0.8%)	2 (0.8%)	9 (0.7%)
**36 months**				
Not completed	199 (41.4%)	231 (48.0%)	102 (42.5%)	532 (44.3%)
In clinic	2 (0.4%)	-	-	2 (0.2%)
Home (Postal)	280 (58.2%)	250 (52.0%)	138 (57.5%)	668 (55.6%)

NB: “Completed” refers to a questionnaire booklet returned to the trial team, regardless of how complete each item, scale or questionnaire.

Appendix C: Supplementary analyses of the Seattle Angina Questionnaire (SAQ-UK)

Table C.1: Overall distribution of observed SAQ-UK scale values, and frequency of floor/ceiling values at baseline, 12 and 36months follow-up. (Ranges 0=worst health, 100=best health)

	3T CMR-Guided Care (n=481)	SPECT-Guided Care (n=481)	NICE CG95 (2010) (n=240)	Total (n=1202)
**Angina Frequency (AF)**				
**Baseline**				
n	476	477	234	1187
Mean (SD)	65.2 (23.88)	66.2 (22.71)	66.2 (22.17)	65.8 (23.07)
Median (Interquartile Range)	70 (50 to 80)	70 (60 to 80)	70 (50 to 80)	70 (50 to 80)
n (%) Min, Max value	9 (1.9%), 33 (6.9%)	3 (0.6%), 34 (7.1%)	2 (0.9%), 16 (6.8%)	14 (1.2%), 83 (7.0%)
**12 months**				
n	325	295	157	777
Mean (SD)	88.4 (19.62)	88.6 (16.33)	85.4 (21.05)	87.9 (18.77)
Median (Interquartile Range)	100 (80 to 100)	100 (80 to 100)	100 (80 to 100)	100 (80 to 100)
n (%) Min, Max value	1 (0.3%), 197 (60.6%)	1 (0.3%), 149 (50.5%)	1 (0.6%), 79 (50.3%)	3 (0.4%), 425 (54.7%)
**36 months**				
n	276	247	132	655
Mean (SD)	90.4 (16.77)	91.0 (14.43)	86.1 (22.67)	89.8 (17.41)
Median (Interquartile Range)	100 (90 to 100)	100 (80 to 100)	100 (80 to 100)	100 (80 to 100)
n (%) Min, Max value	0 (0.0%), 179 (64.9%)	0 (0.0%), 149 (60.3%)	1 (0.8%), 79 (59.8%)	1 (0.2%), 407 (62.1%)
**Angina Stability (AS)**				
**Baseline**				
n	469	468	232	1169
Mean (SD)	45.9 (27.87)	45.5 (26.01)	43.5 (28.13)	45.3 (27.19)
Median (Interquartile Range)	50 (25 to 50)	50 (25 to 50)	50 (25 to 50)	50 (25 to 50)
n (%) Min, Max value	61 (13.0%), 53 (11.3%)	48 (10.3%), 44 (9.4%)	33 (14.2%), 26 (11.2%)	142 (12.1%), 123 (10.5%)
**12 months**				
n	322	296	157	775
Mean (SD)	53.1 (18.57)	59.3 (20.74)	53.3 (20.04)	55.5 (19.92)
Median (Interquartile Range)	50 (50 to 50)	50 (50 to 75)	50 (50 to 50)	50 (50 to 50)
n (%) Min, Max value	8 (2.5%), 29 (9.0%)	4 (1.4%), 49 (16.6%)	5 (3.2%), 17 (10.8%)	17 (2.2%), 95 (12.3%)

	3T CMR-Guided Care (n=481)	SPECT-Guided Care (n=481)	NICE CG95 (2010) (n=240)	Total (n=1202)
**36 months**				
n	275	246	132	653
Mean (SD)	53.9 (17.63)	55.1 (19.94)	50.0 (16.35)	53.6 (18.36)
Median (Interquartile Range)	50 (50 to 50)	50 (50 to 50)	50 (50 to 50)	50 (50 to 50)
n (%) Min, Max value	3 (1.1%), 27 (9.8%)	4 (1.6%), 30 (12.2%)	2 (1.5%), 7 (5.3%)	9 (1.4%), 64 (9.8%)
**Physical Limitation (PL)**				
**Baseline**				
n	463	464	230	1157
Mean (SD)	72.9 (22.33)	72.0 (21.83)	71.2 (24.00)	72.2 (22.46)
Median (Interquartile Range)	77.8 (58.3 to 91.7)	75 (58.3 to 88.9)	77.8 (55.6 to 88.9)	77.8 (58.3 to 91.7)
n (%) Min, Max value	0 (0.0%), 57 (12.3%)	0 (0.0%), 44 (9.5%)	1 (0.4%), 24 (10.4%)	1 (0.1%), 125 (10.8%)
**12 months**				
n	292	271	150	713
Mean (SD)	79.8 (23.28)	79.7 (22.91)	74.0 (26.00)	78.5 (23.82)
Median (Interquartile Range)	88.9 (66.7 to 100)	87.5 (66.7 to 100)	83.3 (55.6 to 97.2)	86.1 (66.7 to 100)
n (%) Min, Max value	1 (0.3%), 94 (32.2%)	2 (0.7%), 84 (31.0%)	0 (0.0%), 33 (22.0%)	3 (0.4%), 211 (29.6%)
**36 months**				
n	256	228	126	610
Mean (SD)	79.9 (24.00)	80.0 (23.63)	72.0 (28.81)	78.3 (25.10)
Median (Interquartile Range)	89.6 (63.9 to 100)	88.9 (66.7 to 100)	83.3 (47.2 to 100)	88.9 (61.1 to 100)
n (%) Min, Max value	0 (0.0%), 92 (35.9%)	0 (0.0%), 71 (31.1%)	1 (0.8%), 36 (28.6%)	1 (0.2%), 199 (32.6%)
**Quality of Life (QoL)**				
**Baseline**				
n	474	475	234	1183
Mean (SD)	52.7 (20.76)	51.4 (20.01)	49.0 (21.33)	51.4 (20.60)
Median (Interquartile Range)	50 (41.7 to 66.7)	50 (41.7 to 66.7)	50 (33.3 to 66.7)	50 (37.5 to 66.7)
n (%) Min, Max value	4 (0.8%), 11 (2.3%)	2 (0.4%), 5 (1.1%)	3 (1.3%), 5 (2.1%)	9 (0.8%), 21 (1.8%)
**12 months**				
n	320	292	155	767
Mean (SD)	73.6 (23.77)	72.9 (20.98)	69.2 (27.07)	72.5 (23.51)
Median (Interquartile Range)	83.3 (58.3 to 91.7)	75 (58.3 to 91.7)	75 (50 to 91.7)	75 (58.3 to 91.7)
n (%) Min, Max value	3 (0.9%), 57 (17.8%)	0 (0.0%), 40 (13.7%)	2 (1.3%), 30 (19.4%)	5 (0.7%), 127 (16.6%)

	3T CMR-Guided Care (n=481)	SPECT-Guided Care (n=481)	NICE CG95 (2010) (n=240)	Total (n=1202)
**36 months**				
n	268	243	127	638
Mean (SD)	76.0 (22.69)	76.3 (21.03)	71.7 (25.33)	75.3 (22.67)
Median (Interquartile Range)	83.3 (58.3 to 91.7)	83.3 (66.7 to 91.7)	83.3 (58.3 to 91.7)	83.3 (58.3 to 91.7)
n (%) Min, Max value	0 (0.0%), 61 (22.8%)	1 (0.4%), 41 (16.9%)	1 (0.8%), 23 (18.1%)	2 (0.3%), 125 (19.6%)
**Treatment Satisfaction (TS)**				
**Baseline**				
n	471	472	233	1176
Mean (SD)	90.6 (12.99)	90.0 (14.61)	89.9 (13.40)	90.2 (13.74)
Median (Interquartile Range)	100 (81.3 to 100)	100 (81.3 to 100)	93.8 (81.3 to 100)	100 (81.3 to 100)
n (%) Min, Max value	0 (0.0%), 244 (51.8%)	0 (0.0%), 250 (53.0%)	0 (0.0%), 113 (48.5%)	0 (0.0%), 607 (51.6%)
**12 months**				
n	322	292	155	769
Mean (SD)	87.2 (19.91)	86.1 (18.29)	85.6 (19.65)	86.5 (19.24)
Median (Interquartile Range)	100 (81.3 to 100)	93.8 (81.3 to 100)	93.8 (81.3 to 100)	100 (81.3 to 100)
n (%) Min, Max value	1 (0.3%), 179 (55.6%)	0 (0.0%), 134 (45.9%)	0 (0.0%), 73 (47.1%)	1 (0.1%), 386 (50.2%)
**36 months**				
n	269	242	129	640
Mean (SD)	88.6 (20.30)	89.4 (16.92)	86.7 (19.97)	88.5 (19.01)
Median (Interquartile Range)	100 (81.3 to 100)	100 (81.3 to 100)	100 (81.3 to 100)	100 (81.3 to 100)
n (%) Min, Max value	2 (0.7%), 169 (62.8%)	0 (0.0%), 137 (56.6%)	0 (0.0%), 71 (55.0%)	2 (0.3%), 377 (58.9%)

Table C2: Comparison of primary and sensitivity mixed effects (random coefficients) modelling of SAQ domains

	CG95 (2010) vs CMR				SPECT vs CMR			
			95% CI				95% CI	
**Domain / Analysis**	**Estimate**	**Standard Error**	**Lower**	**Upper**	**Estimate**	**Standard Error**	**Lower**	**Upper**
**Angina Frequency**								
Primary	-0.023	0.056	-0.133	0.087	0.002	0.044	-0.084	0.088
Baseline*Time	-0.024	0.056	-0.134	0.086	0.002	0.044	-0.084	0.088
Multiple Imputation	-0.048	0.060	-0.167	0.071	0.022	0.049	-0.075	0.119
**Angina Stability**								
Primary	-0.224	0.077	-0.376	-0.073	-0.080	0.064	-0.206	0.046
Baseline*Time	-0.224	0.077	-0.376	-0.073	-0.080	0.064	-0.206	0.046
Multiple Imputation	-0.191	0.072	-0.332	-0.049	-0.067	0.060	-0.184	0.051
Proportional Odds	-0.030	0.010	-0.050	-0.010	-0.010	0.009	-0.027	0.007
**Physical Limitation**								
Primary	-0.072	0.055	-0.180	0.036	0.035	0.045	-0.054	0.124
Baseline*Time	-0.072	0.055	-0.180	0.036	0.034	0.045	-0.055	0.123
Multiple Imputation	-0.048	0.061	-0.169	0.072	0.037	0.049	-0.060	0.134
**Quality of Life**								
Primary	-0.053	0.068	-0.187	0.081	-0.004	0.054	-0.110	0.102
Baseline*Time	-0.065	0.068	-0.198	0.068	-0.014	0.054	-0.120	0.091
Multiple Imputation	-0.043	0.071	-0.183	0.097	0.004	0.056	-0.107	0.114
**Treatment Satisfaction**								
Primary	0.010	0.060	-0.108	0.128	0.106	0.048	0.012	0.200
Baseline*Time	0.008	0.060	-0.109	0.125	0.100	0.048	0.006	0.193
Multiple Imputation	0.027	0.063	-0.097	0.150	0.100	0.049	0.003	0.196

Estimate=Estimated interaction effect between NICE (or SPECT) and time in months. Negative values indicate CMR improving vs comparator, positive values indicate comparator improving vs CMR.

Lower/Upper = Limits of 95% Confidence Interval for the difference

Baseline*Time=Fitting the primary analysis model, with an additional fixed interaction effect for baseline-by-time, allowing patients with different health statuses to have different trajectories during the follow-up.

Proportional Odds=Replacing linear mixed model with an ordinal proportional odds model, modelling the odds of moving up to greater values. Only done for the Angina Stability scale, derived from a single 5-item question. Values <0 represent reduced log-odds per month of moving to higher scores for CG95/SPECT vs CMR, values >0 represent increased log-odds per month of moving up to higher scores vs CMR. Thus the estimate of -0.0301 for Angina Stability in the NICE comparison indicates that the odds of a NICE patient having a higher Angina Stability score change by exp(-0.0301) – 1 = -2.97% per month compared to the CMR arm.

Table C3: Comparison of primary and sensitivity analyses of SAQ domains at 12 and 36 months post-randomisation

			Least-Squares Means				Least Squares Mean differences vs CMR			
					95% CI				95% CI	
**Domain / randomised arm**	**Time**	**Analysis**	**Est.**	**Std Err.**	**Lower**	**Upper**	**Est.**	**Std Err.**	**Lower**	**Upper**
**Angina Frequency**										
CMR (n=325)	12 months	Primary	89.823	1.310	87.253	92.394				
		BL*Time	89.239	1.307	86.674	91.805				
		Mult. Imput.	90.249	1.811	86.698	93.800				
SPECT (n=295)	12 months	Primary	88.202	1.320	85.612	90.793	-1.621	1.404	-4.377	1.134
		BL*Time	87.689	1.317	85.105	90.274	-1.550	1.405	-4.308	1.208
		Mult. Imput.	88.721	1.756	85.278	92.164	-1.528	1.371	-4.219	1.163
CG95 (2010) (n=157)	12 months	Primary	86.612	1.652	83.370	89.855	-3.211	1.701	-6.550	0.128
		BL*Time	86.045	1.646	82.814	89.276	-3.195	1.703	-6.536	0.147
		Mult. Imput.	86.700	2.097	82.587	90.813	-3.549	1.698	-6.882	-0.215
CMR (n=276)	36 months	Primary	90.742	1.314	88.164	93.321				
		BL*Time	90.179	1.312	87.604	92.754				
		Mult. Imput.	91.016	1.798	87.491	94.542				
SPECT (n=247)	36 months	Primary	90.437	1.330	87.827	93.047	-0.305	1.416	-3.083	2.473
		BL*Time	89.977	1.328	87.372	92.582	-0.202	1.412	-2.973	2.569
		Mult. Imput.	91.266	1.808	87.721	94.811	0.250	1.434	-2.568	3.068
CG95 (2010) (n=132)	36 months	Primary	86.488	1.661	83.229	89.747	-4.254	1.715	-7.619	-0.889
		BL*Time	85.960	1.653	82.715	89.204	-4.219	1.710	-7.575	-0.863
		Mult. Imput.	85.999	2.248	81.583	90.415	-5.017	1.802	-8.560	-1.474
**Angina Stability**										
CMR (n=322)	12 months	Primary	53.023	1.338	50.396	55.649				
		BL*Time	53.293	1.339	50.665	55.922				
		Mult. Imput.	55.108	1.714	51.744	58.473				
SPECT (n=296)	12 months	Primary	58.556	1.357	55.894	61.219	5.534	1.582	2.428	8.639
		BL*Time	58.787	1.357	56.123	61.451	5.494	1.583	2.388	8.600
		Mult. Imput.	60.101	1.828	56.509	63.692	4.992	1.563	1.923	8.062
CG95 (2010) (n=157)	12 months	Primary	53.456	1.747	50.029	56.884	0.434	1.914	-3.322	4.190
		BL*Time	53.702	1.746	50.276	57.128	0.409	1.914	-3.349	4.166

			Least-Squares Means				Least Squares Mean differences vs CMR			
					95% CI				95% CI	
Domain / randomised arm	Time	Analysis	Est.	Std Err.	Lower	Upper	Est.	Std Err.	Lower	Upper
		Mult. Imput.	55.479	2.083	51.388	59.571	0.371	1.992	-3.544	4.286
CMR (n=275)	36 months	Primary	53.344	1.345	50.704	55.984				
		BL*Time	53.647	1.351	50.997	56.298				
		Mult. Imput.	54.941	1.671	51.662	58.221				
SPECT (n=246)	36 months	Primary	54.789	1.380	52.081	57.497	1.446	1.602	-1.699	4.590
		BL*Time	55.033	1.386	52.313	57.754	1.386	1.602	-1.759	4.530
		Mult. Imput.	56.069	1.759	52.613	59.525	1.128	1.490	-1.802	4.058
CG95 (2010) (n=132)	36 months	Primary	49.641	1.763	46.181	53.100	-3.703	1.933	-7.497	0.091
		BL*Time	49.892	1.765	46.429	53.356	-3.755	1.934	-7.550	0.040
		Mult. Imput.	51.677	1.991	47.767	55.588	-3.264	1.806	-6.814	0.286
**Physical Limitation**										
CMR (n=292)	12 months	Primary	78.276	1.483	75.365	81.186				
		BL*Time	79.672	1.475	76.778	82.566				
		Mult. Imput.	79.392	2.091	75.289	83.494				
SPECT (n=271)	12 months	Primary	78.093	1.488	75.173	81.014	-0.182	1.558	-3.239	2.875
		BL*Time	79.244	1.476	76.348	82.140	-0.428	1.553	-3.476	2.620
		Mult. Imput.	78.726	2.052	74.701	82.751	-0.665	1.501	-3.611	2.280
CG95 (2010) (n=150)	12 months	Primary	72.073	1.837	68.467	75.679	-6.202	1.872	-9.876	-2.529
		BL*Time	73.377	1.823	69.799	76.955	-6.296	1.865	-9.955	-2.636
		Mult. Imput.	72.797	2.365	68.157	77.437	-6.595	1.812	-10.151	-3.038
CMR (n=256)	36 months	Primary	77.136	1.528	74.137	80.135				
		BL*Time	78.389	1.525	75.397	81.381				
		Mult. Imput.	77.396	2.156	73.164	81.628				
SPECT (n=228)	36 months	Primary	78.353	1.547	75.317	81.388	1.217	1.655	-2.032	4.466
		BL*Time	79.469	1.539	76.449	82.488	1.079	1.654	-2.166	4.325
		Mult. Imput.	78.337	2.179	74.059	82.614	0.941	1.591	-2.185	4.068
CG95 (2010) (n=126)	36 months	Primary	70.453	1.923	66.679	74.227	-6.683	1.991	-10.590	-2.776
		BL*Time	71.706	1.913	67.951	75.461	-6.683	1.988	-10.585	-2.782
		Mult. Imput.	70.940	2.401	66.228	75.653	-6.455	1.941	-10.269	-2.641
**Quality of Life**										

			Least-Squares Means				Least Squares Mean differences vs CMR			
					95% CI				95% CI	
Domain / randomised arm	Time	Analysis	Est.	Std Err.	Lower	Upper	Est.	Std Err.	Lower	Upper
CMR (n=320)	12 months	Primary	73.206	1.583	70.099	76.312				
		BL*Time	72.203	1.574	69.114	75.292				
		Mult. Imput.	71.029	2.356	66.405	75.653				
SPECT (n=292)	12 months	Primary	72.860	1.591	69.738	75.982	-0.346	1.646	-3.576	2.884
		BL*Time	72.078	1.583	68.971	75.184	-0.126	1.645	-3.355	3.103
		Mult. Imput.	71.068	2.274	66.608	75.528	0.039	1.699	-3.298	3.376
CG95 (2010) (n=155)	12 months	Primary	68.943	1.973	65.071	72.815	-4.263	1.992	-8.172	-0.353
		BL*Time	68.101	1.961	64.252	71.951	-4.102	1.991	-8.009	-0.195
		Mult. Imput.	66.689	2.575	61.637	71.742	-4.339	1.959	-8.183	-0.495
CMR (n=268)	36 months	Primary	74.419	1.665	71.152	77.687				
		BL*Time	73.636	1.656	70.385	76.886				
		Mult. Imput.	71.822	2.429	67.056	76.589				
SPECT (n=243)	36 months	Primary	75.247	1.680	71.950	78.545	0.828	1.805	-2.713	4.370
		BL*Time	74.415	1.671	71.136	77.695	0.780	1.796	-2.745	4.305
		Mult. Imput.	72.646	2.466	67.804	77.488	0.824	1.817	-2.747	4.394
CG95 (2010) (n=127)	36 months	Primary	70.054	2.115	65.904	74.205	-4.365	2.192	-8.667	-0.063
		BL*Time	69.139	2.099	65.020	73.258	-4.497	2.182	-8.778	-0.215
		Mult. Imput.	67.175	2.785	61.706	72.645	-4.647	2.176	-8.922	-0.373
**Treatment Satisfaction**										
CMR (n=322)	12 months	Primary	87.597	1.363	84.923	90.272				
		BL*Time	86.351	1.358	83.686	89.016				
		Mult. Imput.	87.333	1.987	83.435	91.231				
SPECT (n=292)	12 months	Primary	86.882	1.375	84.184	89.580	-0.715	1.434	-3.531	2.100
		BL*Time	85.799	1.370	83.111	88.487	-0.552	1.434	-3.366	2.263
		Mult. Imput.	87.446	1.911	83.700	91.192	0.113	1.421	-2.677	2.903
CG95 (2010) (n=155)	12 months	Primary	85.342	1.709	81.987	88.696	-2.256	1.737	-5.665	1.154
		BL*Time	84.184	1.700	80.848	87.520	-2.167	1.737	-5.575	1.241
		Mult. Imput.	85.416	2.205	81.091	89.742	-1.917	1.880	-5.613	1.779
CMR (n=269)	36 months	Primary	87.962	1.426	85.163	90.762				

			Least-Squares Means				Least Squares Mean differences vs CMR			
					95% CI				95% CI	
Domain / randomised arm	Time	Analysis	Est.	Std Err.	Lower	Upper	Est.	Std Err.	Lower	Upper
		BL*Time	86.774	1.424	83.980	89.569				
		Mult. Imput.	87.263	2.012	83.315	91.210				
SPECT (n=242)	36 months	Primary	88.749	1.445	85.914	91.584	0.787	1.556	-2.268	3.841
		BL*Time	87.657	1.442	84.828	90.486	0.883	1.556	-2.170	3.936
		Mult. Imput.	88.533	2.026	84.558	92.507	1.270	1.558	-1.789	4.328
CG95 (2010) (n=129)	36 months	Primary	86.260	1.813	82.702	89.818	-1.702	1.886	-5.403	1.998
		BL*Time	85.154	1.806	81.610	88.698	-1.621	1.884	-5.319	2.078
		Mult. Imput.	85.928	2.401	81.214	90.642	-1.335	2.078	-5.421	2.751

Primary: fixed effects baseline value, time (categorical), randomised arm, arm-by-time interaction, age, sex, randomising centre, pre-test likelihood category. Correlation handled by repeated measures within patients, unstructured covariance pattern.

BL*Time: As for primary, but also including fixed effect for baseline*time interaction.

Appendix D: Supplementary analyses of SF12v2

Table D.1: Overall distribution of observed SF12v2 scale values, and frequency of floor/ceiling values at baseline, 12 and 36months follow-up. (Ranges 0=worst health, 100=best health)

	3T CMR-Guided Care (n=481)	SPECT-Guided Care (n=481)	NICE CG95 (2010) (n=240)	Total (n=1202)
**Body Pain (BP)**				
**Baseline**				
n	478	479	235	1192
Mean (SD)	65.6 (25.75)	64.6 (27.22)	62.9 (26.29)	64.7 (26.45)
Median (Interquartile Range)	75 (50 to 75)	75 (50 to 75)	75 (50 to 75)	75 (50 to 75)
n (%) Min, Max value	14 (2.9%), 94 (19.7%)	22 (4.6%), 96 (20.0%)	8 (3.4%), 39 (16.6%)	44 (3.7%), 229 (19.2%)
**12 months**				
n	331	300	158	789
Mean (SD)	73.0 (28.93)	74.2 (28.10)	70.9 (29.44)	73.0 (28.71)
Median (Interquartile Range)	75 (50 to 100)	75 (50 to 100)	75 (50 to 100)	75 (50 to 100)
n (%) Min, Max value	13 (3.9%), 133 (40.2%)	10 (3.3%), 125 (41.7%)	8 (5.1%), 57 (36.1%)	31 (3.9%), 315 (39.9%)
**36 months**				
n	281	250	137	668
Mean (SD)	75.4 (28.58)	72.1 (29.96)	71.0 (29.59)	73.2 (29.32)
Median (Interquartile Range)	75 (50 to 100)	75 (50 to 100)	75 (50 to 100)	75 (50 to 100)
n (%) Min, Max value	11 (3.9%), 125 (44.5%)	15 (6.0%), 97 (38.8%)	5 (3.6%), 55 (40.1%)	31 (4.6%), 277 (41.5%)
**General Health (GH)**				
**Baseline**				
n	478	479	235	1192
Mean (SD)	53.8 (26.13)	54.0 (25.48)	53.5 (26.80)	53.8 (25.98)
Median (Interquartile Range)	60 (25 to 60)	60 (25 to 60)	60 (25 to 60)	60 (25 to 60)
n (%) Min, Max value	26 (5.4%), 15 (3.1%)	25 (5.2%), 17 (3.5%)	18 (7.7%), 8 (3.4%)	69 (5.8%), 40 (3.4%)
**12 months**				
n	332	301	157	790
Mean (SD)	56.6 (28.07)	57.4 (26.54)	55.4 (26.98)	56.7 (27.25)
Median (Interquartile Range)	60 (25 to 85)	60 (25 to 85)	60 (25 to 85)	60 (25 to 85)
n (%) Min, Max value	24 (7.2%), 15 (4.5%)	15 (5.0%), 12 (4.0%)	13 (8.3%), 6 (3.8%)	52 (6.6%), 33 (4.2%)
**36 months**				
n	280	250	136	666

	3T CMR-Guided Care (n=481)	SPECT-Guided Care (n=481)	NICE CG95 (2010) (n=240)	Total (n=1202)
Mean (SD)	57.5 (28.40)	56.8 (27.21)	56.8 (27.51)	57.1 (27.74)
Median (Interquartile Range)	60 (25 to 85)	60 (25 to 85)	60 (25 to 85)	60 (25 to 85)
n (%) Min, Max value	21 (7.5%), 15 (5.4%)	16 (6.4%), 9 (3.6%)	8 (5.9%), 7 (5.1%)	45 (6.8%), 31 (4.7%)
**Physical Functioning (PF)**				
**Baseline**				
n	478	479	235	1192
Mean (SD)	60.2 (31.05)	58.1 (30.89)	58.2 (31.44)	59.0 (31.05)
Median (Interquartile Range)	50 (50 to 91.1)	50 (45 to 75)	50 (50 to 75)	50 (50 to 75)
n (%) Min, Max value	39 (8.2%), 119 (24.9%)	41 (8.6%), 108 (22.5%)	24 (10.2%), 57 (24.3%)	104 (8.7%), 284 (23.8%)
**12 months**				
n	332	301	158	791
Mean (SD)	62.6 (33.50)	64.0 (33.29)	58.5 (33.06)	62.3 (33.35)
Median (Interquartile Range)	75 (50 to 100)	75 (50 to 100)	50 (25 to 75)	75 (50 to 100)
n (%) Min, Max value	39 (11.7%), 105 (31.6%)	31 (10.3%), 101 (33.6%)	19 (12.0%), 38 (24.1%)	89 (11.3%), 244 (30.8%)
**36 months**				
n	282	250	137	669
Mean (SD)	65.7 (33.26)	63.2 (35.70)	57.1 (36.50)	63.0 (34.95)
Median (Interquartile Range)	75 (50 to 100)	75 (45 to 100)	50 (25 to 100)	75 (45 to 100)
n (%) Min, Max value	26 (9.2%), 99 (35.1%)	33 (13.2%), 91 (36.4%)	25 (18.2%), 39 (28.5%)	84 (12.6%), 229 (34.2%)
**Role Performance (RP)**				
**Baseline**				
n	478	478	234	1190
Mean (SD)	64.3 (26.07)	62.0 (26.11)	63.0 (27.61)	63.1 (26.39)
Median (Interquartile Range)	62.5 (50 to 87.5)	62.5 (50 to 75)	62.5 (50 to 87.5)	62.5 (50 to 78.9)
n (%) Min, Max value	11 (2.3%), 91 (19.0%)	17 (3.6%), 75 (15.7%)	11 (4.7%), 42 (17.9%)	39 (3.3%), 208 (17.5%)
**12 months**				
n	332	300	158	790
Mean (SD)	70.6 (30.18)	70.9 (28.48)	69.0 (29.63)	70.4 (29.41)
Median (Interquartile Range)	75 (50 to 100)	75 (50 to 100)	75 (50 to 100)	75 (50 to 100)
n (%) Min, Max value	19 (5.7%), 113 (34.0%)	12 (4.0%), 98 (32.7%)	7 (4.4%), 49 (31.0%)	38 (4.8%), 260 (32.9%)
**36 months**				
n	282	250	136	668

	3T CMR-Guided Care (n=481)	SPECT-Guided Care (n=481)	NICE CG95 (2010) (n=240)	Total (n=1202)
Mean (SD)	70.2 (29.63)	69.1 (31.03)	67.1 (29.64)	69.2 (30.14)
Median (Interquartile Range)	75 (50 to 100)	75 (50 to 100)	75 (50 to 100)	75 (50 to 100)
n (%) Min, Max value	15 (5.3%), 91 (32.3%)	14 (5.6%), 91 (36.4%)	5 (3.7%), 41 (30.1%)	34 (5.1%), 223 (33.4%)
**Physical Component Summary (PCS)**				
**Baseline**				
n	477	478	235	1190
Mean (SD)	44.2 (9.71)	43.9 (9.25)	44.0 (9.45)	44.1 (9.47)
Median (Interquartile Range)	45.2 (37.4 to 51.7)	44.6 (37.8 to 50.9)	43.9 (38.5 to 51.4)	44.6 (37.8 to 51.4)
n (%) Min, Max value	0 (0.0%), 0 (0.0%)	0 (0.0%), 0 (0.0%)	0 (0.0%), 0 (0.0%)	0 (0.0%), 0 (0.0%)
**12 months**				
n	332	299	157	788
Mean (SD)	46.3 (10.34)	46.6 (10.19)	45.3 (10.22)	46.2 (10.26)
Median (Interquartile Range)	47.7 (40.8 to 55.6)	48.2 (40.6 to 55.4)	46.3 (38.4 to 53.8)	47.6 (39.9 to 55)
n (%) Min, Max value	0 (0.0%), 0 (0.0%)	0 (0.0%), 0 (0.0%)	0 (0.0%), 0 (0.0%)	0 (0.0%), 0 (0.0%)
**36 months**				
n	278	250	137	665
Mean (SD)	46.9 (11.16)	45.8 (11.19)	45.2 (10.99)	46.1 (11.14)
Median (Interquartile Range)	49.5 (40.6 to 55.9)	48.1 (38.3 to 55.6)	46.7 (37.1 to 55.6)	48.5 (38.6 to 55.9)
n (%) Min, Max value	0 (0.0%), 0 (0.0%)	0 (0.0%), 0 (0.0%)	0 (0.0%), 0 (0.0%)	0 (0.0%), 0 (0.0%)
**Mental Health (MH)**				
**Baseline**				
n	477	475	236	1188
Mean (SD)	64.1 (21.17)	63.7 (21.08)	61.5 (23.30)	63.4 (21.58)
Median (Interquartile Range)	62.5 (50 to 75)	62.5 (50 to 75)	62.5 (50 to 75)	62.5 (50 to 75)
n (%) Min, Max value	3 (0.6%), 21 (4.4%)	1 (0.2%), 21 (4.4%)	5 (2.1%), 10 (4.2%)	9 (0.8%), 52 (4.4%)
**12 months**				
n	332	299	157	788
Mean (SD)	65.8 (23.92)	66.8 (21.50)	66.6 (23.03)	66.4 (22.83)
Median (Interquartile Range)	75 (50 to 87.5)	75 (50 to 87.5)	75 (50 to 87.5)	75 (50 to 87.5)
n (%) Min, Max value	5 (1.5%), 33 (9.9%)	2 (0.7%), 23 (7.7%)	2 (1.3%), 10 (6.4%)	9 (1.1%), 66 (8.4%)
**36 months**				
n	278	250	137	665

	3T CMR-Guided Care (n=481)	SPECT-Guided Care (n=481)	NICE CG95 (2010) (n=240)	Total (n=1202)
Mean (SD)	66.8 (23.21)	69.6 (20.45)	66.3 (22.42)	67.7 (22.06)
Median (Interquartile Range)	75 (50 to 87.5)	75 (50 to 87.5)	75 (50 to 87.5)	75 (50 to 87.5)
n (%) Min, Max value	4 (1.4%), 20 (7.2%)	2 (0.8%), 22 (8.8%)	2 (1.5%), 9 (6.6%)	8 (1.2%), 51 (7.7%)
**Role Emotional (RE)**				
**Baseline**				
n	478	478	235	1191
Mean (SD)	79.5 (24.82)	76.2 (26.71)	76.1 (26.70)	77.5 (26.00)
Median (Interquartile Range)	87.5 (62.5 to 100)	87.5 (50 to 100)	75 (50 to 100)	87.5 (62.5 to 100)
n (%) Min, Max value	6 (1.3%), 222 (46.4%)	9 (1.9%), 201 (42.1%)	4 (1.7%), 99 (42.1%)	19 (1.6%), 522 (43.8%)
**12 months**				
n	332	301	158	791
Mean (SD)	79.3 (26.89)	80.8 (25.62)	77.7 (28.47)	79.5 (26.73)
Median (Interquartile Range)	100 (60.7 to 100)	100 (75 to 100)	87.5 (62.5 to 100)	100 (62.5 to 100)
n (%) Min, Max value	10 (3.0%), 169 (50.9%)	4 (1.3%), 151 (50.2%)	5 (3.2%), 77 (48.7%)	19 (2.4%), 397 (50.2%)
**36 months**				
n	282	250	137	669
Mean (SD)	80.2 (25.01)	79.9 (26.17)	76.0 (27.09)	79.2 (25.89)
Median (Interquartile Range)	100 (62.5 to 100)	98 (75 to 100)	75 (50 to 100)	87.5 (62.5 to 100)
n (%) Min, Max value	2 (0.7%), 144 (51.1%)	6 (2.4%), 123 (49.2%)	3 (2.2%), 59 (43.1%)	11 (1.6%), 326 (48.7%)
**Social Functioning (SF)**				
**Baseline**				
n	476	478	236	1190
Mean (SD)	74.0 (28.24)	74.7 (27.40)	71.2 (28.92)	73.7 (28.05)
Median (Interquartile Range)	75 (50 to 100)	75 (50 to 100)	75 (50 to 100)	75 (50 to 100)
n (%) Min, Max value	17 (3.6%), 207 (43.5%)	15 (3.1%), 207 (43.3%)	8 (3.4%), 94 (39.8%)	40 (3.4%), 508 (42.7%)
**12 months**				
n	332	299	158	789
Mean (SD)	77.0 (28.12)	77.1 (27.51)	75.6 (30.12)	76.8 (28.28)
Median (Interquartile Range)	87.5 (50 to 100)	75 (50 to 100)	100 (50 to 100)	100 (50 to 100)
n (%) Min, Max value	13 (3.9%), 166 (50.0%)	9 (3.0%), 149 (49.8%)	6 (3.8%), 81 (51.3%)	28 (3.5%), 396 (50.2%)
**36 months**				
n	278	249	135	662

	3T CMR-Guided Care (n=481)	SPECT-Guided Care (n=481)	NICE CG95 (2010) (n=240)	Total (n=1202)
Mean (SD)	77.1 (27.82)	76.6 (28.97)	73.9 (29.75)	76.2 (28.64)
Median (Interquartile Range)	100 (50 to 100)	100 (50 to 100)	75 (50 to 100)	100 (50 to 100)
n (%) Min, Max value	8 (2.9%), 140 (50.4%)	9 (3.6%), 128 (51.4%)	4 (3.0%), 64 (47.4%)	21 (3.2%), 332 (50.2%)
**Vitality (VT)**				
**Baseline**				
n	474	475	235	1184
Mean (SD)	47.4 (25.62)	44.8 (25.69)	45.7 (26.00)	46.0 (25.73)
Median (Interquartile Range)	50 (25 to 75)	50 (25 to 75)	50 (25 to 75)	50 (25 to 75)
n (%) Min, Max value	47 (9.9%), 13 (2.7%)	59 (12.4%), 14 (2.9%)	27 (11.5%), 6 (2.6%)	133 (11.2%), 33 (2.8%)
**12 months**				
n	327	296	156	779
Mean (SD)	50.5 (26.92)	50.7 (24.86)	51.4 (25.60)	50.7 (25.86)
Median (Interquartile Range)	50 (25 to 75)	50 (25 to 75)	50 (25 to 75)	50 (25 to 75)
n (%) Min, Max value	39 (11.9%), 11 (3.4%)	26 (8.8%), 8 (2.7%)	14 (9.0%), 4 (2.6%)	79 (10.1%), 23 (3.0%)
**36 months**				
n	275	248	137	660
Mean (SD)	50.2 (26.85)	51.1 (25.08)	51.6 (27.15)	50.8 (26.23)
Median (Interquartile Range)	50 (25 to 75)	50 (25 to 75)	50 (25 to 75)	50 (25 to 75)
n (%) Min, Max value	30 (10.9%), 10 (3.6%)	23 (9.3%), 6 (2.4%)	14 (10.2%), 8 (5.8%)	67 (10.2%), 24 (3.6%)
**Mental Component Summary (MCS)**				
**Baseline**				
n	477	475	235	1187
Mean (SD)	49.2 (9.86)	48.6 (10.46)	47.9 (11.10)	48.7 (10.36)
Median (Interquartile Range)	50.7 (43.3 to 57.3)	50.5 (41.6 to 56.8)	49.6 (40.8 to 56.7)	50.5 (41.9 to 56.9)
n (%) Min, Max value	0 (0.0%), 0 (0.0%)	0 (0.0%), 0 (0.0%)	0 (0.0%), 0 (0.0%)	0 (0.0%), 0 (0.0%)
**12 months**				
n	332	299	157	788
Mean (SD)	49.3 (10.83)	49.7 (9.76)	49.7 (10.72)	49.6 (10.40)
Median (Interquartile Range)	52.2 (42.2 to 57.4)	52.3 (43.9 to 57.4)	53 (43.1 to 57.5)	52.3 (43 to 57.4)
n (%) Min, Max value	0 (0.0%), 0 (0.0%)	0 (0.0%), 0 (0.0%)	0 (0.0%), 0 (0.0%)	0 (0.0%), 0 (0.0%)
**36 months**				
n	278	250	137	665

	3T CMR-Guided Care (n=481)	SPECT-Guided Care (n=481)	NICE CG95 (2010) (n=240)	Total (n=1202)
Mean (SD)	49.4 (10.43)	50.4 (9.23)	49.4 (10.20)	49.8 (9.95)
Median (Interquartile Range)	52.3 (41.8 to 57.4)	52.7 (44.7 to 57.4)	51.9 (42.9 to 57.4)	52.4 (43 to 57.4)
n (%) Min, Max value	0 (0.0%), 0 (0.0%)	0 (0.0%), 0 (0.0%)	0 (0.0%), 0 (0.0%)	0 (0.0%), 0 (0.0%)

Table D2: Comparison of primary and sensitivity mixed effects (random coefficients) modelling of SF12v2

	CG95 (2010) vs CMR				SPECT vs CMR			
			95% CI				95% CI	
**Domain / Analysis**	**Estimate**	**Standard Error**	**Lower**	**Upper**	**Estimate**	**Standard Error**	**Lower**	**Upper**
**Body Pain**								
Primary	-0.0777	0.070	-0.2156	0.0602	-0.1058	0.059	-0.2221	0.0106
Baseline*Time	-0.0780	0.070	-0.2161	0.0601	-0.1081	0.059	-0.2246	0.0084
Multiple Imputation	-0.0841	0.078	-0.2368	0.0687	-0.0878	0.063	-0.2116	0.0360
Proportional Odds	-0.0091	0.010	-0.0281	0.0100	-0.0172	0.008	-0.0333	-0.0011
**General Health**								
Primary	-0.0647	0.069	-0.2000	0.0706	0.0048	0.056	-0.1042	0.1138
Baseline*Time	-0.0627	0.069	-0.1978	0.0724	0.0051	0.056	-0.1039	0.1142
Multiple Imputation	-0.0391	0.069	-0.1756	0.0974	0.0119	0.057	-0.1010	0.1248
Proportional Odds	-0.0063	0.010	-0.0256	0.0130	-0.0019	0.008	-0.0181	0.0143
**Mental Component Summary**								
Primary	-0.0057	0.028	-0.0612	0.0498	0.0196	0.023	-0.0260	0.0652
Baseline*Time	-0.0088	0.028	-0.0643	0.0466	0.0152	0.023	-0.0304	0.0607
Multiple Imputation	0.0002	0.028	-0.0554	0.0557	0.0206	0.025	-0.0296	0.0708
**Mental Health**								
Primary	-0.0431	0.063	-0.1674	0.0812	0.0440	0.052	-0.0578	0.1458
Baseline*Time	-0.0438	0.063	-0.1681	0.0805	0.0402	0.052	-0.0614	0.1418
Multiple Imputation	-0.0470	0.065	-0.1758	0.0817	0.0571	0.051	-0.0429	0.1570
**Physical Component Summary**								
Primary	-0.0516	0.025	-0.1006	-0.0025	-0.0162	0.021	-0.0567	0.0243
Baseline*Time	-0.0512	0.025	-0.1002	-0.0022	-0.0170	0.021	-0.0575	0.0236
Multiple Imputation	-0.0513	0.027	-0.1043	0.0016	-0.0149	0.023	-0.0597	0.0299
**Physical Functioning**								
Primary	-0.2242	0.083	-0.3862	-0.0623	-0.0355	0.065	-0.1637	0.0926
Baseline*Time	-0.2254	0.083	-0.3874	-0.0633	-0.0414	0.065	-0.1698	0.0871
Multiple Imputation	-0.2296	0.079	-0.3851	-0.0741	-0.0335	0.067	-0.1645	0.0975
**Role Emotional**								
Primary	-0.0610	0.071	-0.2007	0.0787	0.0024	0.060	-0.1161	0.1209
Baseline*Time	-0.0680	0.071	-0.2083	0.0722	-0.0055	0.061	-0.1246	0.1136
Multiple Imputation	-0.0160	0.078	-0.1690	0.1370	0.0168	0.061	-0.1041	0.1377
**Role Performance**								
Primary	-0.0996	0.070	-0.2379	0.0387	0.0191	0.059	-0.0976	0.1358

	CG95 (2010) vs CMR				SPECT vs CMR			
			95% CI				95% CI	
**Domain / Analysis**	**Estimate**	**Standard Error**	**Lower**	**Upper**	**Estimate**	**Standard Error**	**Lower**	**Upper**
Baseline*Time	-0.0979	0.070	-0.2360	0.0402	0.0154	0.060	-0.1015	0.1323
Multiple Imputation	-0.0810	0.069	-0.2165	0.0546	0.0138	0.062	-0.1079	0.1354
**Social Functioning**								
Primary	-0.0829	0.076	-0.2324	0.0666	-0.0297	0.064	-0.1561	0.0967
Baseline*Time	-0.0857	0.076	-0.2354	0.0641	-0.0319	0.064	-0.1584	0.0946
Multiple Imputation	-0.0663	0.087	-0.2386	0.1060	-0.0384	0.072	-0.1805	0.1036
Proportional Odds	-0.0094	0.010	-0.0281	0.0094	-0.0054	0.008	-0.0212	0.0104
**Vitality**								
Primary	-0.0255	0.074	-0.1707	0.1197	0.0737	0.060	-0.0447	0.1922
Baseline*Time	-0.0246	0.074	-0.1699	0.1207	0.0740	0.061	-0.0451	0.1932
Multiple Imputation	-0.0222	0.079	-0.1775	0.1332	0.0791	0.061	-0.0402	0.1985
Proportional Odds	0.0006	0.009	-0.0170	0.0183	0.0078	0.008	-0.0071	0.0227

Estimate=Estimated interaction effect between NICE (or SPECT) and time in months. Negative values indicate CMR improving vs comparator, positive values indicate comparator improving vs CMR.

Lower/Upper = Limits of 95% Confidence Interval for the difference

Baseline*Time=Fitting the primary analysis model, with an additional fixed interaction effect for baseline-by-time, allowing patients with different health statuses to have different trajectories during the follow-up.

Proportional Odds=Replacing linear mixed model with an ordinal proportional odds model, modelling the odds of moving up to greater values. Only done for the Body Pain, General Health, Social Functioning and Vitality scales, derived from a single 5-item question. Values <0 represent reduced log-odds per month of moving to higher scores for CG95/SPECT vs CMR, values >0 represent increased log-odds per month of moving up to higher scores vs CMR. Thus the estimate of = -0.0172 for Body Pain in the SPECT comparison indicates that the odds of a SPECT patient having a higher Body Pain score change by exp(-0.0172) - 1 = -1.71% per month compared to the CMR arm.

Table D3: Comparison of primary and sensitivity Repeated Measures (covariance pattern) models of SF12v2 domains at 12 and 36 months post-randomisation

			Least-Squares Means				Least Squares Mean differences vs CMR			
					95% CI				95% CI	
Domain / randomised arm	Time	Analysis	Est.	Std Err	Lower	Upper	Est.	Std Err	Lower	Upper
**Body Pain**										
CMR (n=331)	12 months	Primary	71.244	1.763	67.785	74.703				
		BL*Time	71.268	1.762	67.809	74.727				
		Mult. Imput.	70.088	2.510	65.166	75.010				
SPECT (n=300)	12 months	Primary	72.525	1.771	69.049	76.001	1.281	1.862	-2.373	4.935
		BL*Time	72.514	1.771	69.039	75.990	1.246	1.862	-2.408	4.901
		Mult. Imput.	71.038	2.483	66.169	75.907	0.950	1.927	-2.835	4.735
CG95 (2010) (n=158)	12 months	Primary	69.102	2.217	64.751	73.452	-2.142	2.263	-6.583	2.299
		BL*Time	69.111	2.217	64.761	73.461	-2.157	2.263	-6.598	2.284
		Mult. Imput.	68.125	2.839	62.557	73.693	-1.963	2.196	-6.270	2.344
CMR (n=281)	36 months	Primary	72.534	1.849	68.906	76.162				
		BL*Time	72.555	1.849	68.926	76.184				
		Mult. Imput.	70.607	2.528	65.649	75.565				
SPECT (n=250)	36 months	Primary	70.213	1.872	66.539	73.887	-2.321	2.033	-6.311	1.669
		BL*Time	70.200	1.872	66.526	73.874	-2.355	2.034	-6.347	1.638
		Mult. Imput.	68.404	2.648	63.210	73.598	-2.203	2.178	-6.487	2.081
CG95 (2010) (n=137)	36 months	Primary	68.521	2.347	63.915	73.126	-4.013	2.455	-8.831	0.805
		BL*Time	68.542	2.347	63.936	73.149	-4.013	2.456	-8.832	0.807
		Mult. Imput.	66.198	2.995	60.321	72.075	-4.409	2.605	-9.529	0.711
**General Health**										
CMR (n=332)	12 months	Primary	56.038	1.494	53.106	58.971				
		BL*Time	56.040	1.494	53.107	58.972				
		Mult. Imput.	56.907	2.089	52.811	61.002				
SPECT (n=301)	12 months	Primary	55.894	1.503	52.944	58.844	-0.144	1.577	-3.239	2.951
		BL*Time	55.885	1.503	52.935	58.835	-0.155	1.577	-3.250	2.940
		Mult. Imput.	56.919	2.115	52.772	61.066	0.012	1.589	-3.108	3.133
CG95 (2010) (n=157)	12 months	Primary	53.386	1.885	49.685	57.086	-2.653	1.920	-6.421	1.116
		BL*Time	53.368	1.886	49.667	57.069	-2.672	1.920	-6.440	1.097

			Least-Squares Means				Least Squares Mean differences vs CMR			
					95% CI				95% CI	
Domain / randomised arm	Time	Analysis	Est.	Std Err	Lower	Upper	Est.	Std Err	Lower	Upper
		Mult. Imput.	54.756	2.404	50.040	59.472	-2.150	1.958	-5.994	1.693
CMR (n=280)	36 months	Primary	55.716	1.634	52.508	58.923				
		BL*Time	55.767	1.634	52.560	58.974				
		Mult. Imput.	56.521	2.252	52.102	60.940				
SPECT (n=250)	36 months	Primary	54.805	1.661	51.546	58.065	-0.910	1.847	-4.535	2.714
		BL*Time	54.835	1.661	51.576	58.095	-0.932	1.846	-4.555	2.691
		Mult. Imput.	55.906	2.225	51.541	60.272	-0.615	1.809	-4.168	2.939
CG95 (2010) (n=136)	36 months	Primary	53.555	2.098	49.438	57.673	-2.160	2.231	-6.538	2.218
		BL*Time	53.634	2.099	49.515	57.753	-2.133	2.231	-6.511	2.245
		Mult. Imput.	55.036	2.580	49.974	60.097	-1.485	2.220	-5.846	2.875
**Mental Component Summary**										
CMR (n=332)	12 months	Primary	49.673	0.630	48.436	50.909				
		BL*Time	49.683	0.630	48.446	50.919				
		Mult. Imput.	49.168	0.901	47.400	50.935				
SPECT (n=299)	12 months	Primary	50.411	0.636	49.163	51.658	0.738	0.683	-0.603	2.079
		BL*Time	50.419	0.636	49.171	51.667	0.736	0.684	-0.606	2.079
		Mult. Imput.	49.937	0.908	48.155	51.719	0.770	0.697	-0.598	2.138
CG95 (2010) (n=157)	12 months	Primary	49.965	0.802	48.392	51.539	0.293	0.831	-1.337	1.923
		BL*Time	49.970	0.802	48.396	51.543	0.287	0.831	-1.344	1.917
		Mult. Imput.	49.344	1.037	47.309	51.378	0.176	0.858	-1.510	1.862
CMR (n=278)	36 months	Primary	49.412	0.653	48.130	50.693				
		BL*Time	49.508	0.652	48.229	50.788				
		Mult. Imput.	49.126	0.925	47.310	50.941				
SPECT (n=250)	36 months	Primary	50.931	0.662	49.632	52.231	1.519	0.727	0.093	2.946
		BL*Time	50.926	0.660	49.630	52.221	1.417	0.725	-0.005	2.839
		Mult. Imput.	50.665	0.936	48.828	52.502	1.539	0.793	-0.021	3.100
CG95 (2010) (n=137)	36 months	Primary	49.667	0.830	48.037	51.296	0.255	0.876	-1.464	1.974
		BL*Time	49.684	0.828	48.060	51.309	0.176	0.872	-1.536	1.888
		Mult. Imput.	49.345	1.092	47.201	51.489	0.219	0.928	-1.606	2.045

			Least-Squares Means				Least Squares Mean differences vs CMR			
					95% CI				95% CI	
Domain / randomised arm	Time	Analysis	Est.	Std Err	Lower	Upper	Est.	Std Err	Lower	Upper
**Mental Health**										
CMR (n=332)	12 months	Primary	65.783	1.399	63.038	68.528				
		BL*Time	65.794	1.399	63.048	68.539				
		Mult. Imput.	64.988	1.997	61.072	68.905				
SPECT (n=299)	12 months	Primary	66.961	1.413	64.187	69.734	1.178	1.530	-1.825	4.180
		BL*Time	66.958	1.414	64.184	69.732	1.164	1.531	-1.840	4.168
		Mult. Imput.	66.151	1.970	62.288	70.014	1.162	1.546	-1.873	4.198
CG95 (2010) (n=157)	12 months	Primary	65.782	1.786	62.277	69.288	-0.001	1.860	-3.652	3.650
		BL*Time	65.784	1.787	62.278	69.291	-0.009	1.861	-3.662	3.643
		Mult. Imput.	64.646	2.313	60.107	69.184	-0.342	1.879	-4.032	3.347
CMR (n=278)	36 months	Primary	65.995	1.436	63.176	68.813				
		BL*Time	66.056	1.436	63.237	68.875				
		Mult. Imput.	65.708	1.997	61.791	69.626				
SPECT (n=250)	36 months	Primary	69.426	1.457	66.567	72.284	3.431	1.600	0.290	6.572
		BL*Time	69.433	1.456	66.576	72.291	3.377	1.600	0.237	6.517
		Mult. Imput.	69.303	1.973	65.433	73.174	3.595	1.527	0.597	6.592
CG95 (2010) (n=137)	36 months	Primary	65.434	1.827	61.849	69.019	-0.561	1.928	-4.344	3.222
		BL*Time	65.492	1.827	61.907	69.077	-0.564	1.927	-4.345	3.217
		Mult. Imput.	64.700	2.239	60.308	69.092	-1.008	1.898	-4.736	2.719
**Physical Component Summary**										
CMR (n=332)	12 months	Primary	45.836	0.555	44.747	46.925				
		BL*Time	45.836	0.555	44.747	46.925				
		Mult. Imput.	46.623	0.797	45.059	48.187				
SPECT (n=299)	12 months	Primary	46.161	0.559	45.065	47.258	0.325	0.581	-0.814	1.465
		BL*Time	46.156	0.559	45.060	47.252	0.320	0.581	-0.820	1.459
		Mult. Imput.	46.972	0.804	45.395	48.549	0.349	0.557	-0.744	1.441
CG95 (2010) (n=157)	12 months	Primary	44.502	0.698	43.133	45.871	-1.334	0.706	-2.720	0.053
		BL*Time	44.516	0.697	43.147	45.884	-1.321	0.706	-2.707	0.065
		Mult. Imput.	45.501	0.916	43.705	47.298	-1.122	0.698	-2.493	0.249

			Least-Squares Means				Least Squares Mean differences vs CMR			
					95% CI				95% CI	
**Domain / randomised arm**	**Time**	**Analysis**	**Est.**	**Std Err**	**Lower**	**Upper**	**Est.**	**Std Err**	**Lower**	**Upper**
CMR (n=278)	36 months	Primary	45.967	0.620	44.750	47.184				
		BL*Time	45.968	0.620	44.751	47.186				
		Mult. Imput.	46.590	0.847	44.928	48.251				
SPECT (n=250)	36 months	Primary	45.264	0.630	44.028	46.501	-0.702	0.705	-2.085	0.681
		BL*Time	45.260	0.630	44.023	46.497	-0.708	0.705	-2.092	0.676
		Mult. Imput.	45.902	0.911	44.113	47.691	-0.688	0.740	-2.143	0.766
CG95 (2010) (n=137)	36 months	Primary	43.705	0.797	42.142	45.268	-2.261	0.850	-3.930	-0.592
		BL*Time	43.724	0.797	42.160	45.288	-2.244	0.851	-3.914	-0.575
		Mult. Imput.	44.463	1.017	42.467	46.459	-2.127	0.863	-3.823	-0.432
**Physical Functioning**										
CMR (n=332)	12 months	Primary	60.088	1.901	56.356	63.819				
		BL*Time	60.119	1.901	56.388	63.851				
		Mult. Imput.	61.328	2.676	56.082	66.574				
SPECT (n=301)	12 months	Primary	62.905	1.916	59.144	66.665	2.817	2.018	-1.144	6.778
		BL*Time	62.847	1.916	59.087	66.607	2.728	2.018	-1.233	6.688
		Mult. Imput.	63.583	2.689	58.310	68.855	2.254	1.980	-1.632	6.141
CG95 (2010) (n=158)	12 months	Primary	56.132	2.400	51.422	60.841	-3.956	2.453	-8.770	0.859
		BL*Time	56.154	2.399	51.446	60.862	-3.966	2.452	-8.778	0.846
		Mult. Imput.	57.595	3.069	51.574	63.615	-3.734	2.534	-8.711	1.244
CMR (n=282)	36 months	Primary	61.741	2.027	57.764	65.719				
		BL*Time	61.758	2.029	57.776	65.740				
		Mult. Imput.	63.310	2.708	58.000	68.621				
SPECT (n=250)	36 months	Primary	62.245	2.062	58.198	66.293	0.504	2.266	-3.943	4.951
		BL*Time	62.224	2.064	58.174	66.274	0.466	2.270	-3.989	4.921
		Mult. Imput.	63.294	2.860	57.683	68.906	-0.016	2.230	-4.396	4.364
CG95 (2010) (n=137)	36 months	Primary	53.080	2.590	47.997	58.162	-8.662	2.732	-14.023	-3.300
		BL*Time	53.127	2.592	48.040	58.214	-8.631	2.734	-13.995	-3.266
		Mult. Imput.	54.447	3.182	48.206	60.689	-8.863	2.632	-14.029	-3.696
**Role Emotional**										

			Least-Squares Means				Least Squares Mean differences vs CMR			
					95% CI				95% CI	
Domain / randomised arm	Time	Analysis	Est.	Std Err	Lower	Upper	Est.	Std Err	Lower	Upper
CMR (n=332)	12 months	Primary	79.859	1.656	76.609	83.110				
		BL*Time	79.787	1.656	76.537	83.037				
		Mult. Imput.	78.958	2.394	74.262	83.653				
SPECT (n=301)	12 months	Primary	82.806	1.669	79.531	86.082	2.947	1.773	-0.532	6.427
		BL*Time	82.877	1.669	79.602	86.151	3.090	1.775	-0.393	6.573
		Mult. Imput.	81.650	2.326	77.089	86.212	2.692	1.773	-0.789	6.173
CG95 (2010) (n=158)	12 months	Primary	79.258	2.095	75.147	83.370	-0.601	2.154	-4.828	3.627
		BL*Time	79.267	2.095	75.157	83.378	-0.519	2.154	-4.748	3.709
		Mult. Imput.	77.543	2.695	72.256	82.831	-1.414	2.269	-5.872	3.044
CMR (n=282)	36 months	Primary	80.165	1.737	76.757	83.574				
		BL*Time	80.402	1.736	76.994	83.809				
		Mult. Imput.	79.056	2.529	74.092	84.021				
SPECT (n=250)	36 months	Primary	81.873	1.767	78.406	85.339	1.707	1.933	-2.087	5.502
		BL*Time	81.803	1.763	78.342	85.263	1.401	1.933	-2.393	5.194
		Mult. Imput.	81.050	2.453	76.236	85.864	1.993	1.972	-1.883	5.870
CG95 (2010) (n=137)	36 months	Primary	76.949	2.213	72.606	81.292	-3.216	2.329	-7.787	1.354
		BL*Time	76.943	2.208	72.610	81.276	-3.459	2.325	-8.021	1.104
		Mult. Imput.	76.124	2.817	70.594	81.654	-2.932	2.429	-7.708	1.844
**Role Performance**										
CMR (n=332)	12 months	Primary	70.748	1.775	67.265	74.231				
		BL*Time	70.736	1.775	67.253	74.219				
		Mult. Imput.	74.126	2.517	69.191	79.061				
SPECT (n=300)	12 months	Primary	71.806	1.787	68.299	75.312	1.057	1.849	-2.571	4.686
		BL*Time	71.780	1.787	68.273	75.287	1.044	1.850	-2.587	4.674
		Mult. Imput.	75.285	2.494	70.395	80.175	1.159	1.857	-2.487	4.805
CG95 (2010) (n=158)	12 months	Primary	68.123	2.223	63.760	72.486	-2.625	2.246	-7.033	1.783
		BL*Time	68.139	2.223	63.776	72.502	-2.597	2.247	-7.006	1.812
		Mult. Imput.	72.025	2.827	66.481	77.568	-2.101	2.334	-6.686	2.483
CMR (n=282)	36 months	Primary	69.313	1.891	65.602	73.024				

			Least-Squares Means				Least Squares Mean differences vs CMR			
					95% CI				95% CI	
Domain / randomised arm	Time	Analysis	Est.	Std Err	Lower	Upper	Est.	Std Err	Lower	Upper
		BL*Time	69.325	1.891	65.613	73.037				
		Mult. Imput.	72.551	2.605	67.443	77.659				
SPECT (n=250)	36 months	Primary	69.738	1.921	65.967	73.509	0.425	2.082	-3.660	4.511
		BL*Time	69.697	1.921	65.926	73.468	0.372	2.083	-3.716	4.460
		Mult. Imput.	72.844	2.721	67.505	78.183	0.294	2.094	-3.821	4.409
CG95 (2010) (n=136)	36 months	Primary	64.447	2.405	59.727	69.168	-4.866	2.514	-9.799	0.068
		BL*Time	64.501	2.406	59.778	69.223	-4.824	2.514	-9.758	0.109
		Mult. Imput.	68.379	3.021	62.451	74.306	-4.172	2.557	-9.197	0.854
**Social Functioning**										
CMR (n=332)	12 months	Primary	76.718	1.755	73.274	80.162				
		BL*Time	76.717	1.755	73.272	80.161				
		Mult. Imput.	76.006	2.566	70.974	81.038				
SPECT (n=299)	12 months	Primary	76.300	1.767	72.833	79.768	-0.418	1.853	-4.055	3.219
		BL*Time	76.306	1.767	72.837	79.774	-0.411	1.854	-4.050	3.227
		Mult. Imput.	75.816	2.553	70.808	80.823	-0.190	1.956	-4.032	3.651
CG95 (2010) (n=158)	12 months	Primary	75.409	2.208	71.075	79.742	-1.309	2.251	-5.726	3.108
		BL*Time	75.409	2.209	71.074	79.744	-1.308	2.252	-5.727	3.111
		Mult. Imput.	74.463	3.028	68.519	80.407	-1.543	2.439	-6.335	3.249
CMR (n=278)	36 months	Primary	75.234	1.896	71.514	78.954				
		BL*Time	75.393	1.895	71.674	79.111				
		Mult. Imput.	75.217	2.683	69.954	80.481				
SPECT (n=249)	36 months	Primary	75.049	1.925	71.271	78.827	-0.185	2.124	-4.353	3.984
		BL*Time	75.067	1.922	71.295	78.840	-0.325	2.121	-4.487	3.837
		Mult. Imput.	74.616	2.665	69.388	79.844	-0.602	2.161	-4.849	3.646
CG95 (2010) (n=135)	36 months	Primary	73.742	2.426	68.981	78.502	-1.492	2.567	-6.530	3.546
		BL*Time	73.752	2.422	68.999	78.505	-1.641	2.563	-6.669	3.388
		Mult. Imput.	73.650	3.156	67.454	79.847	-1.567	2.661	-6.799	3.664
**Vitality**										
CMR (n=327)	12 months	Primary	50.747	1.592	47.623	53.871				
		BL*Time	50.819	1.593	47.693	53.944				

			Least-Squares Means				Least Squares Mean differences vs CMR			
					95% CI				95% CI	
Domain / randomised arm	Time	Analysis	Est.	Std Err	Lower	Upper	Est.	Std Err	Lower	Upper
		Mult. Imput.	52.280	2.176	48.012	56.547				
SPECT (n=296)	12 months	Primary	52.654	1.611	49.493	55.815	1.907	1.741	-1.510	5.324
		BL*Time	52.580	1.612	49.418	55.743	1.762	1.744	-1.661	5.184
		Mult. Imput.	54.107	2.237	49.718	58.496	1.827	1.748	-1.605	5.259
CG95 (2010) (n=156)	12 months	Primary	51.135	2.028	47.154	55.115	0.387	2.111	-3.755	4.530
		BL*Time	51.183	2.028	47.202	55.164	0.364	2.111	-3.778	4.506
		Mult. Imput.	52.951	2.625	47.797	58.104	0.671	2.250	-3.752	5.093
CMR (n=275)	36 months	Primary	49.353	1.664	46.087	52.619				
		BL*Time	49.347	1.667	46.076	52.619				
		Mult. Imput.	50.866	2.265	46.423	55.309				
SPECT (n=248)	36 months	Primary	53.211	1.693	49.888	56.534	3.858	1.877	0.175	7.540
		BL*Time	53.220	1.694	49.895	56.545	3.873	1.883	0.178	7.567
		Mult. Imput.	54.926	2.295	50.423	59.429	4.060	1.880	0.365	7.755
CG95 (2010) (n=137)	36 months	Primary	50.046	2.118	45.889	54.203	0.693	2.250	-3.722	5.108
		BL*Time	50.061	2.119	45.902	54.220	0.714	2.251	-3.703	5.131
		Mult. Imput.	51.661	2.612	46.534	56.787	0.795	2.280	-3.688	5.277

Primary: fixed effects baseline value, time (categorical), randomised arm, arm-by-time interaction, age, sex, randomising centre, pre-test likelihood category. Correlation handled by repeated measures within patients, unstructured covariance pattern.

BL*Time: As for primary, but also including fixed effect for baseline*time interaction.

Appendix E: Supplementary analyses of Euroqol EQ-5D

Table E1: Overall distribution of observed EQ-5D-3L and EQ-5D-5L scale values, and frequency of floor/ceiling values at baseline, 12 and 36months follow-up. (Ranges: for -3L, -0.594= worst health. For -5L -0.281 = worst health. For both, 0=unconscious, 1=best health)

	3T CMR-Guided Care (n=481)	SPECT-Guided Care (n=481)	NICE CG95 (2010) (n=240)	Total (n=1202)
**EQ-5D-3L Utility**				
**Baseline**				
n	466	468	230	1164
Mean (SD)	0.757 (0.222)	0.743 (0.227)	0.728 (0.250)	0.746 (0.230)
Median (Interquartile Range)	0.796 (0.691 to 0.883)	0.760 (0.689 to 0.848)	0.743 (0.656 to 0.883)	0.760 (0.689 to 0.850)
n (%) Min, Max value	0 (0.0%), 113 (24.2%)	0 (0.0%), 103 (22.0%)	0 (0.0%), 57 (24.8%)	0 (0.0%), 273 (23.5%)
**12 months**				
n	322	295	155	772
Mean (SD)	0.785 (0.260)	0.776 (0.247)	0.746 (0.304)	0.774 (0.265)
Median (Interquartile Range)	0.812 (0.691 to 1.000)	0.796 (0.691 to 1.000)	0.796 (0.689 to 1.000)	0.796 (0.691 to 1.000)
n (%) Min, Max value	0 (0.0%), 130 (40.4%)	0 (0.0%), 106 (35.9%)	0 (0.0%), 59 (38.1%)	0 (0.0%), 295 (38.2%)
**36 months**				
n	275	247	133	655
Mean (SD)	0.791 (0.250)	0.756 (0.283)	0.741 (0.274)	0.767 (0.268)
Median (Interquartile Range)	0.848 (0.691 to 1.000)	0.796 (0.689 to 1.000)	0.796 (0.656 to 1.000)	0.796 (0.689 to 1.000)
n (%) Min, Max value	0 (0.0%), 113 (41.1%)	0 (0.0%), 86 (34.8%)	0 (0.0%), 42 (31.6%)	0 (0.0%), 241 (36.8%)
**EQ-5D-5L Utility**				
**Baseline**				
n	468	469	231	1168
Mean (SD)	0.843 (0.161)	0.831 (0.175)	0.817 (0.191)	0.833 (0.173)
Median (Interquartile Range)	0.879 (0.778 to 0.937)	0.859 (0.777 to 0.937)	0.859 (0.733 to 0.937)	0.861 (0.777 to 0.937)
n (%) Min, Max value	0 (0.0%), 98 (20.9%)	0 (0.0%), 96 (20.5%)	0 (0.0%), 50 (21.6%)	0 (0.0%), 244 (20.9%)
**12 months**				
n	320	295	156	771
Mean (SD)	0.845 (0.205)	0.843 (0.191)	0.829 (0.212)	0.841 (0.201)
Median (Interquartile Range)	0.922 (0.782 to 1.000)	0.892 (0.777 to 1.000)	0.887 (0.777 to 1.000)	0.896 (0.777 to 1.000)
n (%) Min, Max value	0 (0.0%), 111 (34.7%)	0 (0.0%), 96 (32.5%)	0 (0.0%), 50 (32.1%)	0 (0.0%), 257 (33.3%)

	3T CMR-Guided Care (n=481)	SPECT-Guided Care (n=481)	NICE CG95 (2010) (n=240)	Total (n=1202)
**36 months**				
n	278	246	135	659
Mean (SD)	0.850 (0.199)	0.826 (0.219)	0.808 (0.221)	0.833 (0.212)
Median (Interquartile Range)	0.916 (0.779 to 1.000)	0.887 (0.777 to 1.000)	0.859 (0.745 to 1.000)	0.892 (0.777 to 1.000)
n (%) Min, Max value	0 (0.0%), 110 (39.6%)	0 (0.0%), 70 (28.5%)	0 (0.0%), 38 (28.1%)	0 (0.0%), 218 (33.1%)

Table E2: Comparison of primary and sensitivity analyses of EQ5D-3L and EQ-5D-5L utilities analysed using mixed effects (random coefficients) models

	CG95 (2010) vs CMR				SPECT vs CMR			
			95% CI				95% CI	
**Domain / Analysis**	**Estimate**	**Standard Error**	**Lower**	**Upper**	**Estimate**	**Standard Error**	**Lower**	**Upper**
**3L Utility**								
Primary	-0.0009	0.0007	-0.0022	0.0004	-0.0007	0.0006	-0.0018	0.0004
Baseline*Time	-0.0009	0.0007	-0.0022	0.0004	-0.0007	0.0006	-0.0018	0.0004
Multiple Imputation	-0.0007	0.0007	-0.0021	0.0007	-0.0006	0.0006	-0.0017	0.0006
Proportional Odds	-0.0108	0.0107	-0.0317	0.0101	-0.0097	0.0118	-0.0328	0.0134
**5L Utility**								
Primary	-0.0009	0.0005	-0.0019	0.0001	-0.0006	0.0004	-0.0014	0.0001
Baseline*Time	-0.0009	0.0005	-0.0019	0.0001	-0.0006	0.0004	-0.0014	0.0001
Multiple Imputation	-0.0009	0.0005	-0.0019	0.0002	-0.0006	0.0004	-0.0015	0.0003
Proportional Odds	-0.0157	0.0066	-0.0286	-0.0029	-0.0111	0.0049	-0.0208	-0.0015

Estimate=Estimated interaction effect between NICE (or SPECT) and time in months. Negative values indicate CMR improving vs comparator, positive values indicate comparator improving vs CMR.

Lower/Upper = Limits of 95% Confidence Interval for the difference

Baseline*Time=Fitting the primary analysis model, with an additional fixed interaction effect for baseline-by-time, allowing patients with different health statuses to have different trajectories during the follow-up.

Proportional Odds=Replacing linear mixed model with an ordinal proportional odds model, modelling the odds of moving up to greater values. Performed as an additional analysis, in light of the skewed distribution. Values <0 represent reduced log-odds per month of moving to higher scores for CG95/SPECT vs CMR, values >0 represent increased log-odds per month of moving up to higher scores vs CMR. Thus the estimate of -0.01571 for -5L Utility in the NICE comparison indicates that the odds of a NICE patient having a higher -5L Utility change by exp(-0.0157) -1 = -1.56% per month compared to the CMR arm.

Table E3: Comparison of primary and sensitivity Repeated Measures (covariance pattern) modelling of EQ-5D-3L and EQ-5D-5L at 12 and 36months post-randomisation

			Least-Squares Means				Least Squares Mean differences vs CMR			
					95% CI				95% CI	
Domain / randomised arm	Time	Analysis	Est.	Std Err.	Lower	Upper	Est.	Std Err.	Lower	Upper
**3L Utility**										
CMR (n=322)	12 months	Primary	0.786	0.016	0.754	0.818				
		BL*Time	0.791	0.016	0.759	0.823				
		Mult. Imput.	0.795	0.023	0.749	0.841				
SPECT (n=295)	12 months	Primary	0.779	0.016	0.747	0.811	-0.007	0.017	-0.041	0.028
		BL*Time	0.782	0.016	0.750	0.814	-0.008	0.017	-0.043	0.026
		Mult. Imput.	0.784	0.023	0.739	0.830	-0.011	0.018	-0.046	0.024
CG95 (2010) (n=155)	12 months	Primary	0.746	0.021	0.706	0.787	-0.039	0.021	-0.081	0.002
		BL*Time	0.751	0.020	0.711	0.791	-0.040	0.021	-0.081	0.002
		Mult. Imput.	0.763	0.027	0.710	0.816	-0.032	0.021	-0.074	0.010
CMR (n=275)	36 months	Primary	0.782	0.017	0.749	0.814				
		BL*Time	0.786	0.017	0.754	0.818				
		Mult. Imput.	0.794	0.024	0.747	0.841				
SPECT (n=247)	36 months	Primary	0.761	0.017	0.728	0.794	-0.021	0.018	-0.056	0.015
		BL*Time	0.765	0.017	0.732	0.797	-0.021	0.018	-0.057	0.014
		Mult. Imput.	0.772	0.024	0.725	0.818	-0.022	0.018	-0.057	0.013
CG95 (2010) (n=133)	36 months	Primary	0.737	0.021	0.695	0.778	-0.045	0.022	-0.088	-0.002
		BL*Time	0.741	0.021	0.700	0.782	-0.045	0.022	-0.088	-0.002
		Mult. Imput.	0.755	0.028	0.700	0.811	-0.039	0.022	-0.082	0.005
**5L (Crosswalk) Utility**										
CMR (n=320)	12 months	Primary	0.801	0.014	0.773	0.829				
		BL*Time	0.804	0.014	0.776	0.831				
		Mult. Imput.	0.791	0.020	0.752	0.831				
SPECT (n=295)	12 months	Primary	0.796	0.014	0.768	0.824	-0.005	0.015	-0.034	0.025
		BL*Time	0.798	0.014	0.770	0.826	-0.006	0.015	-0.035	0.024
		Mult. Imput.	0.786	0.020	0.746	0.825	-0.006	0.015	-0.036	0.024
CG95 (2010) (n=156)	12 months	Primary	0.769	0.018	0.734	0.804	-0.032	0.018	-0.068	0.003

			Least-Squares Means				Least Squares Mean differences vs CMR			
					95% CI				95% CI	
Domain / randomised arm	Time	Analysis	Est.	Std Err.	Lower	Upper	Est.	Std Err.	Lower	Upper
		BL*Time	0.771	0.018	0.736	0.806	-0.033	0.018	-0.068	0.003
		Mult. Imput.	0.763	0.024	0.716	0.811	-0.028	0.019	-0.066	0.010
CMR (n=278)	36 months	Primary	0.801	0.015	0.772	0.830				
		BL*Time	0.803	0.015	0.775	0.832				
		Mult. Imput.	0.787	0.020	0.747	0.827				
SPECT (n=246)	36 months	Primary	0.775	0.015	0.746	0.804	-0.026	0.016	-0.057	0.005
		BL*Time	0.777	0.015	0.748	0.806	-0.026	0.016	-0.058	0.005
		Mult. Imput.	0.765	0.020	0.725	0.805	-0.022	0.015	-0.052	0.007
CG95 (2010) (n=135)	36 months	Primary	0.756	0.018	0.720	0.792	-0.045	0.019	-0.083	-0.007
		BL*Time	0.758	0.018	0.722	0.795	-0.045	0.019	-0.083	-0.007
		Mult. Imput.	0.752	0.026	0.702	0.803	-0.035	0.021	-0.076	0.006
**5L Utility**										
CMR (n=320)	12 months	Primary	0.854	0.012	0.831	0.877				
		BL*Time	0.856	0.012	0.834	0.879				
		Mult. Imput.	0.836	0.017	0.803	0.870				
SPECT (n=295)	12 months	Primary	0.854	0.012	0.831	0.877	0.000	0.012	-0.024	0.024
		BL*Time	0.856	0.012	0.833	0.879	-0.000	0.012	-0.024	0.024
		Mult. Imput.	0.837	0.018	0.802	0.872	0.001	0.013	-0.025	0.027
CG95 (2010) (n=156)	12 months	Primary	0.837	0.015	0.808	0.866	-0.017	0.015	-0.046	0.012
		BL*Time	0.839	0.015	0.811	0.868	-0.017	0.015	-0.046	0.012
		Mult. Imput.	0.829	0.019	0.792	0.867	-0.007	0.014	-0.035	0.022
CMR (n=278)	36 months	Primary	0.851	0.012	0.826	0.875				
		BL*Time	0.852	0.012	0.828	0.876				
		Mult. Imput.	0.832	0.017	0.798	0.866				
SPECT (n=246)	36 months	Primary	0.837	0.013	0.813	0.862	-0.013	0.014	-0.040	0.013
		BL*Time	0.840	0.012	0.815	0.864	-0.013	0.014	-0.039	0.014
		Mult. Imput.	0.818	0.018	0.783	0.854	-0.014	0.014	-0.041	0.014
CG95 (2010) (n=135)	36 months	Primary	0.815	0.016	0.784	0.845	-0.036	0.016	-0.068	-0.004
		BL*Time	0.817	0.016	0.786	0.847	-0.036	0.016	-0.068	-0.003
		Mult. Imput.	0.802	0.021	0.761	0.842	-0.030	0.016	-0.062	0.002

Primary: fixed effects baseline value, time (categorical), randomised arm, arm-by-time interaction, age, sex, randomising centre, pre-test likelihood category. Correlation handled by repeated measures within patients, unstructured covariance pattern.

BL*Time: As for primary, but also including fixed effect for baseline*time interaction.
